# Comparative Performance Analysis of Planar MIM Diodes with Novel Electrode–Insulator Material Combinations for LWIR Energy Harvesting

**DOI:** 10.3390/ma19173791

**Published:** 2026-09-06

**Authors:** Rocco Citroni, Luca Balestreri, Fabio Mangini, Fabrizio Frezza

**Affiliations:** 1 Department of Information Engineering, Electronics and Telecommunications (DIET), “La Sapienza” University of Rome, 00184 Rome, Italy; luca.balestreri@uniroma1.it (L.B.); fabrizio.frezza@uniroma1.it (F.F.); 2 Department of Engineering, Niccolò Cusano University, 00166 Rome, Italy; fabio.mangini@unicusano.it

**Keywords:** MIM tunneling diodes, LWIR rectennas, energy harvesting, simmons model, FOM, barrier height engineering

## Abstract

**Highlights:**

**What are the main findings?**

- A theoretical quantum-tunneling analysis identified unexplored MIM diode architectures based on transition-metal dichalcogenides, conductive carbides and nitrides, and rare-earth oxide/oxyhalide tunnel barriers for LWIR rectification.
- The TaS_2_/LaOBr/V MIM diode achieved the best overall performance, exhibiting an asymmetry factor above 2.5 × 10^5^, a nonlinearity of approximately unity, a zero-bias responsivity of 10 V^−1^, and the highest current density at 300 K.

**What are the implications of the main findings?**

- The proposed material platform provides design guidelines for optimizing tunnel-barrier height and material selection in high-performance MIM tunneling diodes.
- The TaS_2_/LaOBr/V architecture represents a promising candidate for efficient zero-bias LWIR rectennas and infrared energy-harvesting systems.

**Abstract:**

Metal–Insulator–Metal (MIM) tunneling diodes are among the most promising rectifying devices for long-wave infrared (LWIR) rectenna systems due to their ultrafast response and zero-bias operation. However, their performance is strongly dependent on the choice of electrode and dielectric materials, making the identification of optimal material combinations a key challenge. To address this issue, this theoretical study presents a numerical investigation of a new class of MIM diodes based on a quantum-mechanical tunneling framework. Novel combinations of transition-metal dichalcogenides (NbS_2_, VSe_2_, and TaS_2_) as anode materials (M1), conductive carbides and nitrides (Mo_2_C, VN, and V) as cathode materials (M2), and rare-earth oxide and oxyhalide compounds (Sc_2_O_3_, LaOF, and LaOBr) as tunnel barriers (I) were selected through an extensive literature survey. These materials were combined to design previously unexplored MIM architectures for LWIR rectification. The electrical transport and rectification properties were evaluated using the Simmons tunneling model by calculating the current density–voltage (J–V) and current–voltage (I–V) characteristics, together with key figures of merit (FOMs), including zero-bias resistance, asymmetry factor, nonlinearity, and responsivity, at room temperature (300 K). The effects of tunnel barrier height and dielectric properties on device performance were systematically investigated. Among all the investigated architectures, the TaS_2_/LaOBr/V MIM diode exhibited the most promising overall performance, achieving an asymmetry factor exceeding 2.5 × 10^5^, a nonlinearity factor of 1, and a zero-bias responsivity of 10 V^−1^ at 300 K. Furthermore, this structure demonstrated the highest current density and the most favorable I–V characteristics among the proposed material combinations. These results identify the TaS_2_/LaOBr/V material system as a promising candidate for high-performance LWIR energy harvesting applications, owing to its optimized tunnel barrier height, which promotes efficient electron tunneling while maintaining excellent rectification properties.

## 1. Introduction

Long-wave infrared (LWIR) radiation represents a naturally available and ubiquitous form of electromagnetic energy emitted as thermal radiation by terrestrial objects, including the Earth’s surface, at near-ambient temperatures (~300 K). At these temperatures, thermal radiation is predominantly distributed across the infrared spectrum, with a significant portion lying within the LWIR region. In particular, wavelengths around 10.6 μm, corresponding to approximately 28.3 THz, are of particular interest for infrared energy-harvesting applications. Accordingly, 10.6 μm (28.3 THz) was selected as the target operating wavelength in this study. Unlike solar energy harvesting, which is strongly dependent on illumination conditions, atmospheric conditions, and the day–night cycle, terrestrial infrared radiation is continuously emitted by objects at temperatures above absolute zero, offering the possibility of harvesting thermal electromagnetic energy independently of sunlight availability. The continuous and ubiquitous nature of thermal infrared radiation has stimulated the development of emerging technologies for LWIR energy harvesting and infrared detection. Among these approaches, infrared rectenna systems, also referred to as infrared rectifying antennas (IRAs), have emerged as a promising platform for the direct conversion of high-frequency electromagnetic radiation into direct-current (DC) electrical power. An infrared rectenna combines an electromagnetic receiving antenna with an ultrafast rectifying element. The antenna captures the incident infrared radiation and generates an alternating-current (AC) voltage at the nanoscale junction, while the rectifier converts this high-frequency AC signal into a usable DC output. Consequently, the rectifying diode represents one of the most critical components of the overall system, since its electrical characteristics ultimately determine the efficiency and feasibility of electromagnetic-to-DC energy conversion. Metal–Insulator–Metal (MIM) tunneling diodes have attracted considerable interest as candidate rectifying elements for LWIR and terahertz (THz) applications because of their quantum-mechanical transport mechanism and potentially ultrafast response. Unlike conventional semiconductor-based rectifiers, whose high-frequency performance is constrained by carrier mobility, carrier transit time, and junction capacitance, MIM diodes rely on direct electron tunneling through an ultrathin insulating barrier. Because tunneling occurs across a nanometer-scale dielectric without requiring thermionic carrier transport over a conventional semiconductor junction, MIM structures can exhibit extremely short response times and potentially support operation from the THz to the infrared regime and, theoretically, beyond 100 THz [1,2,3,4]. This intrinsic high-frequency capability makes MIM tunneling diodes particularly attractive for rectification at infrared frequencies, where conventional semiconductor rectifiers face increasingly stringent speed limitations. The rectification properties of an MIM diode are strongly determined by the electronic properties of the metal–insulator interfaces and, consequently, by the shape and asymmetry of the tunnel barrier. In particular, the work functions of the two electrodes and the electron affinity of the insulating material determine the theoretical barrier heights at the two interfaces. For an electrode with work function ΦM and an insulating layer with electron affinity χ, the corresponding electron tunneling barrier can be approximately described by the difference between ΦM and χ. Therefore, selecting electrode materials with substantially different work functions, combined with an appropriately engineered dielectric electron affinity, provides a means of controlling the barrier asymmetry and, consequently, the tunneling probability under forward and reverse bias. A lower barrier at one interface can facilitate electron tunneling and increase the forward current, whereas a higher barrier at the opposite interface can suppress reverse current. This asymmetric energy landscape is fundamental to achieving efficient rectification. For practical LWIR rectification, a high-performance MIM diode should simultaneously provide sufficiently high current density, pronounced I–V asymmetry, strong nonlinear response, high responsivity, low turn-on voltage (TOV), and efficient operation at zero or near-zero bias. These requirements are particularly important for infrared energy harvesting because the rectenna is expected to operate with extremely small voltage and current levels generated by weak thermal radiation. In this operating regime, the diode must provide efficient nonlinear rectification without relying on a large externally applied DC bias. The optimization of the electrode–insulator combination is therefore a key design strategy for tailoring the tunnel barrier and improving the overall rectification performance. Despite the considerable theoretical potential of MIM tunneling diodes, their practical realization remains challenging. Major technological limitations include the fabrication of uniform and defect-free ultrathin dielectric barriers, precise control of nanometer-scale junction dimensions, control of electrode–insulator interface quality, and reproducible integration of emerging materials [5,6]. At such small thicknesses, even minor variations in dielectric thickness or interface properties can produce significant changes in tunneling probability because of the strong exponential dependence of tunneling current on barrier width and height. These effects can consequently modify the effective barrier profile, parasitic capacitance, leakage current, and overall electrical performance. Therefore, the identification and theoretical evaluation of suitable material combinations represent an important preliminary step toward the development of experimentally realizable high-performance MIM rectifiers. In this context, the present theoretical study investigates a new class of MIM tunneling diodes through a systematic comparison of three planar device architectures based on different electrode–insulator material combinations. The proposed structures employ emerging transition-metal dichalcogenides, namely NbS2, VSe2, and TaS2, as anodes (M1); conductive carbide and nitride materials, namely Mo2C, VN, and V, as cathodes (M2); and rare-earth oxide and oxyhalide materials, namely Sc2O3, LaOF, and LaOBr, as ultrathin insulating tunnel barriers (I). The material combinations were selected through a systematic review and critical analysis of state-of-the-art LWIR MIM diode technologies, considering the reported work functions, electron affinities, theoretical barrier heights, and relevant rectification figures of merit. Particular attention is given to the intrinsic effect of the electrode–insulator material combination on the tunnel barrier profile. To enable a meaningful comparison among the three architectures, the dielectric thickness is kept constant, while the electrode and dielectric materials are varied. The theoretical barrier heights at the metal–insulator interfaces are estimated from the difference between the electrode work function (ΦM) and the dielectric electron affinity (χ). This approach provides a first-order description of the energy-band alignment and enables the resulting barrier asymmetry to be related directly to the expected tunneling behavior. The resulting material-dependent barrier profiles are subsequently analyzed to determine how differences in barrier height and asymmetry influence electron tunneling probability and the resulting rectification characteristics. The electrical response of the proposed structures is evaluated using the Simmons quantum-mechanical tunneling model. The model is employed to calculate the current density–voltage (J–V) characteristics and corresponding current–voltage (I–V) response at room temperature (300 K). From these characteristics, key rectification figures of merit (FOM) are extracted, including the zero-bias differential resistance, current asymmetry, nonlinearity, and responsivity. These parameters provide complementary information on the suitability of each material combination for high-frequency rectification and near-zero-bias infrared energy harvesting. The results demonstrate that engineering the electrode work functions and dielectric electron affinity can substantially modify the tunnel barrier profile and, consequently, the electrical response of the MIM diode. In particular, the combination of a low barrier at one electrode–insulator interface and a comparatively high barrier at the opposite interface promotes preferential tunneling in one bias direction, thereby enhancing current asymmetry and nonlinear rectification. Among the investigated architectures, TaS2/LaOBr/V exhibits the most favorable overall combination of asymmetry, nonlinearity, and responsivity, highlighting the potential of advanced electrode–insulator material systems for high-performance LWIR rectification [7,8]. Although the present results are obtained from a theoretical model, they provide a physically motivated framework for identifying promising material combinations and establishing design guidelines prior to experimental fabrication and characterization. Overall, this work demonstrates that the performance of MIM tunneling diodes can be systematically tailored through material and energy-barrier engineering. The proposed analysis provides insight into the relationship between electrode work function, dielectric electron affinity, tunnel-barrier asymmetry, and rectification performance, offering useful design guidelines for the development of next-generation MIM diodes intended for LWIR energy harvesting, infrared rectennas, and ultrafast electromagnetic-to-DC energy conversion. The remainder of this paper is organized as follows. Section 2 introduces the theoretical framework of quantum tunneling in MIM structures. Section 3 describes the investigated device architectures and simulation methodology. Section 4 presents the calculated electrical characteristics and extracted rectification figures of merit. Finally, Section 5 discusses the main conclusions and future perspectives.

## 2. Theoretical Background

The electrical performance of the proposed MIM tunneling diodes was evaluated from their current–voltage (I–V) characteristics, from which the key rectification FOMs were extracted. These parameters provide a quantitative assessment of the rectification capability and potential for converting high-frequency AC signals into DC power. The resulting FOMs were systematically compared with those reported for state-of-the-art LWIR MIM diodes, as summarized in Table 1 and Table 2 [6]. Although all three proposed diodes are governed by the same Simmons tunneling model and therefore exhibit similar qualitative behavior, their quantitative electrical performance is primarily determined by the height and asymmetry of the interfacial tunneling barriers. These characteristics arise from the different electrode work functions and dielectric electron affinities, which define the electronic band alignment at the metal–insulator interfaces and, consequently, influence the tunneling probability and nonlinear charge transport [6]. Because the tunneling probability depends exponentially on the effective barrier parameters, even relatively small variations in barrier height or profile can produce significant differences in the J–V characteristics, differential resistance, nonlinearity, and responsivity. Since the barrier thickness is identical for all three structures, the observed differences in electrical performance can primarily be attributed to their distinct electronic band alignments and resulting interfacial barrier profiles. Accordingly, the electronic band alignments and tunneling barriers governing the operation of each proposed MIM diode are illustrated below.

### Simmons Model-Based Quantum Engineering of Asymmetric MIM Tunneling Diodes

In the proposed MIM diode structures, electron transport is dominated by quantum-mechanical tunneling through the ultrathin insulating barrier. To accurately describe this transport mechanism and calculate the tunneling current density J(V) as a function of the applied bias voltage V, the asymmetric MIM junction is modeled using the Simmons tunneling formalism [22,23]. The corresponding expression for the tunneling current density is expressed as follows:

(1) $$J(V)=\frac{q}{2\pi hd^{2}}\left[(\Phi -\frac{qV}{2})e^{-Ad\sqrt{\Phi -\frac{qV}{2}}}-(\Phi +\frac{qV}{2})e^{-Ad\sqrt{\Phi +\frac{qV}{2}}}\right]$$

where $A=\frac{4\pi \sqrt{2m}}{h},$ q is the electron charge, h is Planck’s constant, m is the effective electron mass, d is the tunnel barrier thickness, and Φ represents the effective barrier height at the metal–insulator interface. The effective electron mass m was selected according to the transport properties of the insulating material, based on available literature/theoretical data. It was treated as a material-dependent parameter rather than a fitting parameter. The tunnel barrier thickness d was chosen to ensure sufficient quantum tunneling while balancing high tunneling probability against the resulting increase in junction capacitance. The barrier height is determined by the electronic properties of the electrode and dielectric materials and can be approximated as the difference between the metal work function and the electron affinity of the insulating layer [22,23]. The resulting work function difference between the electrodes produces the asymmetric barriers required for rectification. All calculations were performed at 300 K. Under these conditions, the dominant contribution to electron transport across the MIM junction is quantum-mechanical tunneling, whose transmission probability is only weakly dependent on temperature. Temperature effects on the calculated current are therefore mainly associated with the thermal broadening of the Fermi–Dirac distributions, while the temperature dependence of the material parameters is comparatively minor. These parameters were therefore selected according to the physical properties of each material combination.

**Figure 1:**
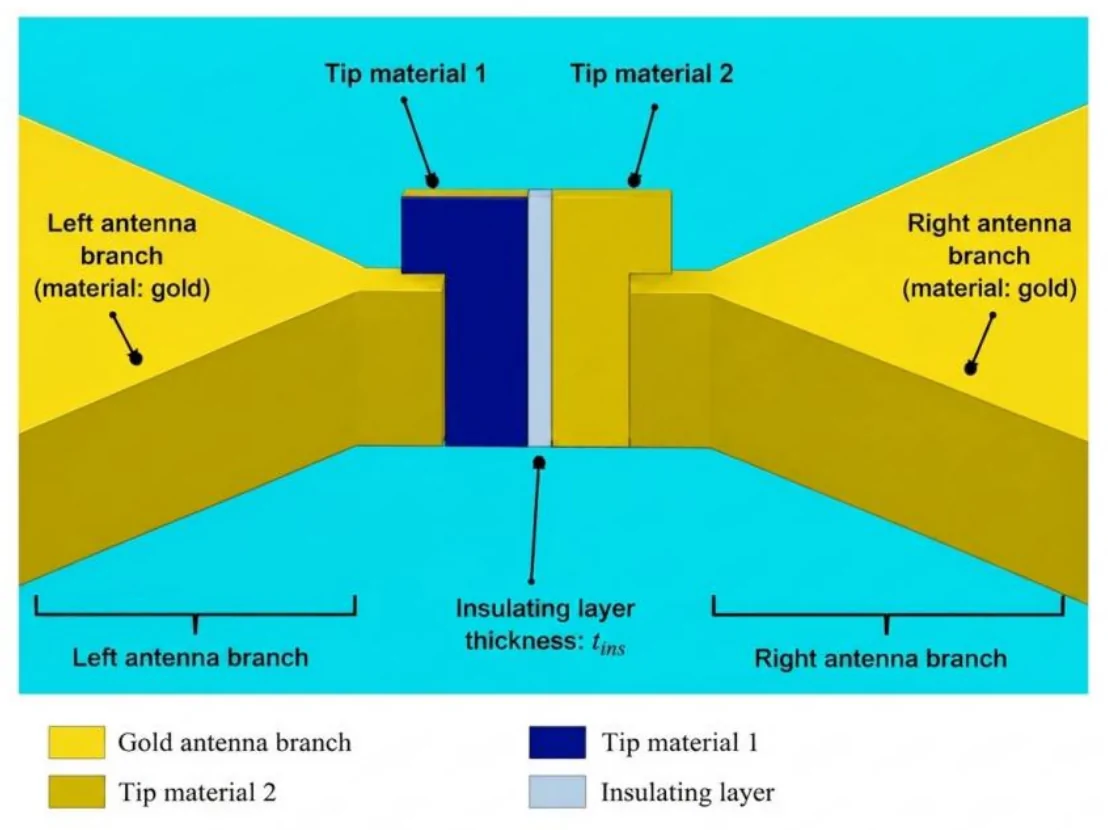
Detail of an asymmetric MIM diode integrated with an antenna structure.

The effective barrier height and dielectric thickness are key parameters governing the tunneling probability and, consequently, the electrical performance of the MIM diode. In particular, because the tunneling process exhibits an exponential dependence on the barrier thickness, even small variations in d can significantly affect the current density, junction resistance, and rectification efficiency. Unlike conventional semiconductor diodes, MIM structures can exhibit finite current flow even under zero applied bias. This behavior originates from the intrinsic asymmetry of the tunneling potential barrier, which arises from the different work functions of the electrodes and the unequal barrier heights at the two metal–insulator interfaces. The total current flowing through the MIM junction can be calculated from the current density as follows [23]:

(2) I(V)=J(V)⋅A

where A represents the effective overlap area between the top and bottom electrodes defining the active tunneling region [23]. The resulting current–voltage (I–V) characteristic provides direct information on the rectification capability of the device. For an asymmetric MIM diode, the forward $I_{F}(V_{D})\;=\;f_{\mathrm{ASYM}}\;e^{\alpha V_{D}}$, and reverse $I_{R}(V_{D})\;=\;-e^{-\alpha V_{D}}$ currents are governed by the different tunneling probabilities across the two barrier configurations [15]. Here, V_D_ is the applied diode voltage, while α is the fitting parameter extracted from the semi-logarithmic I–V slope, defined as the asymmetry factor. The asymmetry factor $f_{\mathrm{ASYM}\;}={\;10}^{3.3137(\Psi_{M1}-\Psi_{M2})}$ depends primarily on the work function difference between the two metallic electrodes [24]. A larger work function difference enhances electron injection under forward bias while suppressing reverse current flow, thereby improving rectification performance. The numerical simulations were performed in the MATLAB(R2025b) environment using a general-purpose computational framework, in which only the material-dependent parameters were modified for each investigated MIM junction configuration. Figure 1 schematically illustrates the structure of an asymmetric MIM diode integrated with an antenna. The device consists of two opposing antenna arms that gradually taper toward the central nanogap, forming the two metallic electrodes of the MIM junction. The antenna arms are made of gold and serve both as electromagnetic receiving elements and as electrical connections to the MIM diode. At the center of the structure, the two metallic electrodes terminate in different tip materials, forming an asymmetric MIM configuration. A thin insulating layer is positioned between the two tips, defining the MIM tunneling barrier, with a thickness t_ins_. The insulating layer electrically separates the two metallic electrodes while allowing electron transport through quantum tunneling. The resulting structure consists of Metal 1/Insulator/Metal 2, with the two metals potentially having different work functions. This asymmetric electrode configuration is particularly relevant for controlling the tunneling barrier heights and enhancing the rectifying and nonlinear behavior of the MIM diode. The antenna arms concentrate the incident electromagnetic field at the central junction, where the resulting high-frequency voltage is applied across the insulating barrier. Consequently, the antenna and MIM diode are electromagnetically and electrically coupled, enabling the structure to operate as a rectifying element for LWIR radiation.

For each MIM configuration, the relevant physical parameters, including the metal work functions, the dielectric constant and electron affinity of the insulating layer, were selected according to the intrinsic properties of the constituent materials and are summarized in the corresponding sections.

## 3. Results and Discussion of MIM Diode Performance

### 3.1. Simulation Setup and Material Parameters

All investigated devices share the same multilayer platform, in which the active MIM tunneling junction is integrated within a common stack. From bottom to top, the structure consists of a 50 nm Au back reflector, which acts as a ground plane to enhance radiation efficiency and reduce substrate losses; a 500 nm high-resistivity Si substrate (εr = 11.9, ρ = 10,000  Ωcm); a 300 nm SiO_2_ matching layer (εr = 3.9, tanδ = 0.001) for impedance matching and improved THz-wave coupling; and a 3 nm Cr adhesion layer to ensure mechanical stability [25,26]. The material selection followed a combined physical design strategy aimed at engineering the interfacial barrier height and asymmetry through appropriate electrode–insulator combinations. The insulating materials were selected based on their dielectric permittivity and electron affinity. A low permittivity εr reduces the junction capacitance and increases the RC cut-off frequency, while an appropriate electron affinity relative to the electrode work functions enables asymmetric interfacial barriers. In particular, when the electron affinity is close to the work function of one electrode, a lower barrier is formed at that interface, while the different work function of the opposite electrode produces a higher barrier, thereby promoting asymmetric tunneling and rectification. The electrodes were therefore selected to provide a sufficiently large work function difference to enhance barrier asymmetry and nonlinear transport. Accordingly, the material selection considered the dielectric permittivity, electron affinity, electrode work functions, and resulting band alignment and barrier profiles. A common nominal barrier thickness of 2 nm was adopted for all architectures to eliminate thickness as an independent variable and isolate the effects of the material-dependent electronic properties. This thickness lies within the ultrathin regime where quantum-mechanical tunneling remains appreciable and represents a realistic nanoscale dielectric thickness. The 2 nm thickness was therefore used as a common basis for the comparative numerical analysis.

### 3.2. NbS_2_-Sc_2_O_3_-Mo_2_C Diode

The first investigated MIM architecture comprises an asymmetric niobium disulfide–scandium oxide–molybdenum carbide (NbS_2_/Sc_2_O_3_/Mo_2_C) trilayer designed for operation at 28.3 THz. NbS_2_ and Mo_2_C were selected as the top and bottom electrodes, respectively, owing to their different work functions, which, together with the electron affinity of Sc_2_O_3_, establish asymmetric tunnel barriers that favor quantum-mechanical tunneling and rectification. The ultrathin Sc_2_O_3_ insulating layer was selected for its suitable electron affinity, moderate dielectric permittivity (εr = 13), low leakage current, and favorable zero-bias rectification characteristics, making it suitable for LWIR energy-harvesting applications.

**Figure 2:**
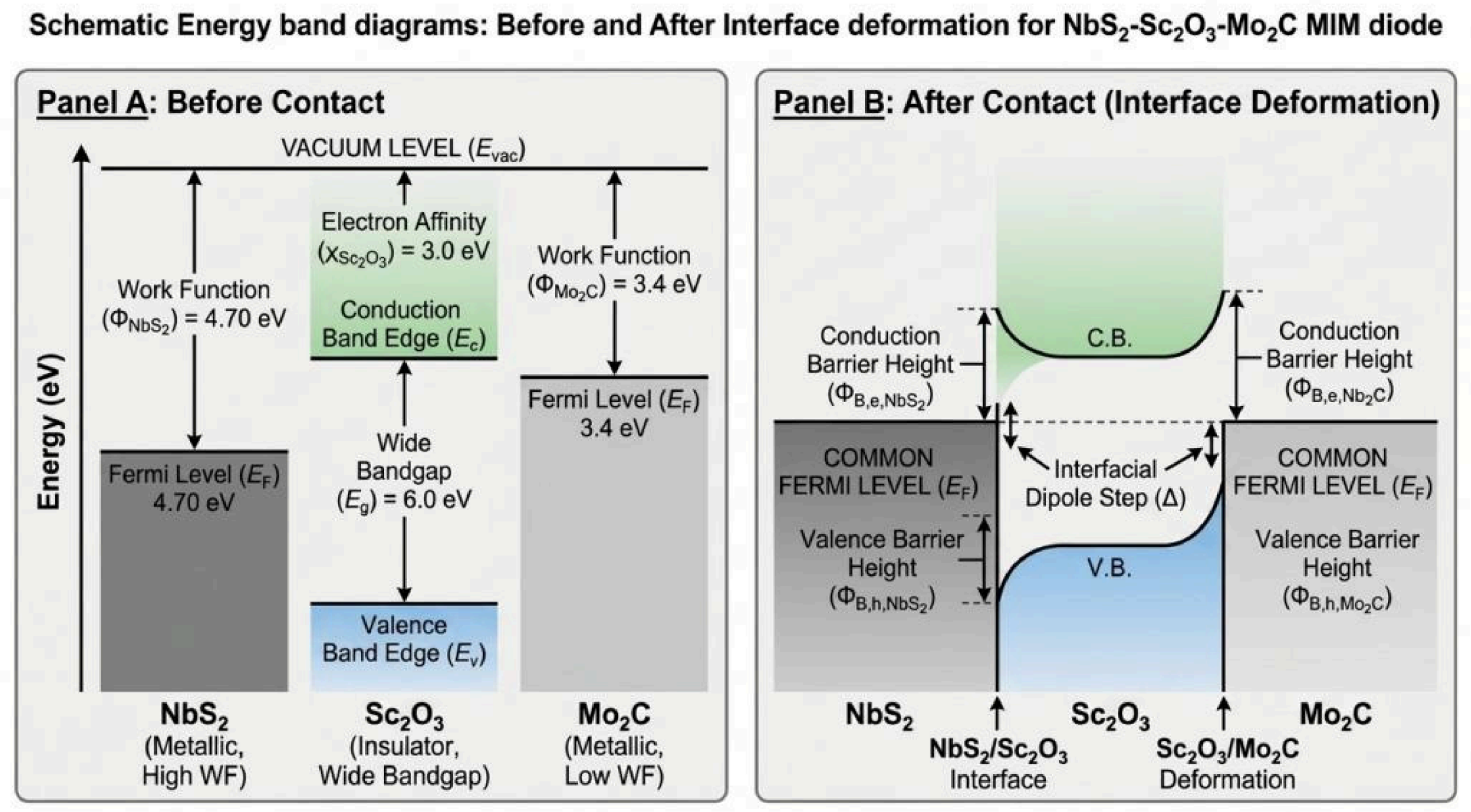
Schematic energy band diagrams of the NbS_2_-Sc_2_O_3_-Mo_2_C structure. (**A**) Before contact: energy band alignment based on vacuum-level alignment (E_vac_), using work functions of 4.70 eV for NbS_2_ and 3.40 eV for Mo_2_C, and an electron affinity of 3.00 eV for Sc2O3. (**B**) After contact (interface formation): realistic band diagram under thermodynamic equilibrium, showing Fermi level (E_F_) alignment, band bending within the Sc_2_O_3_ insulator, and the presence of interfacial dipole steps (∆).

The junction consists of 100 nm-thick NbS_2_ and Mo_2_C electrodes separated by a 2 nm Sc_2_O_3_ tunnel barrier, with an active area of 21 × 21 nm^2^ (441 nm^2^). This nanoscale junction minimizes the junction capacitance and provides an RC time constant compatible with 28.3 THz operation. Under applied bias, electrons tunnel through the Sc_2_O_3_ barrier, while the asymmetric potential profile promotes forward tunneling and suppresses the reverse current, producing the nonlinear I–V response required for efficient rectification. The resulting combination of barrier asymmetry, low junction capacitance, and enhanced zero-bias responsivity makes the NbS_2_/Sc_2_O_3_/Mo_2_C architecture a promising candidate for passive LWIR energy harvesting and self-powered sensing applications. Figure 2 illustrates the evolution of the energy-band alignment of the NbS_2_/Sc_2_O_3_/Mo_2_C heterostructure before and after interface formation. Before contact (Figure 2A), the electronic energy levels of the individual components are referenced to a common vacuum level (Evac) according to the Schottky–Mott model. NbS_2_ exhibits a relatively high work function of 4.70 eV, whereas Mo_2_C acts as the lower-work function electrode, with a work function of 3.40 eV. The Sc_2_O_3_ dielectric layer has an electron affinity of 3.00 eV. In this uncoupled state, the ideal electron Schottky barrier heights at the left and right interfaces are estimated as 1.70 eV for NbS_2_/Sc_2_O_3_ and 0.40 eV for Mo_2_C/Sc_2_O_3_, respectively. Upon interface formation (Figure 2B), charge redistribution occurs across the junctions to establish thermodynamic equilibrium, enforcing a constant Fermi level (E_F_) throughout the system. Due to the significant work function asymmetry (1.30 eV), electrons transfer from the NbS_2_ side toward Mo_2_C. This causes pronounced potential drop and band bending across the ultra-thin LaOF dielectric barrier. Additionally, local charge rearrangement and metal-induced gap states (MIGS) create an interfacial dipole step (∆), modifying the effective Schottky barrier heights (ΦB,n NbS_2_ and ΦB,h Mo_2_C relative to the ideal Schottky–Mott predictions.

The main geometrical, material, and electrical parameters are summarized in Table 3 [27,28,29].

**Figure 3:**
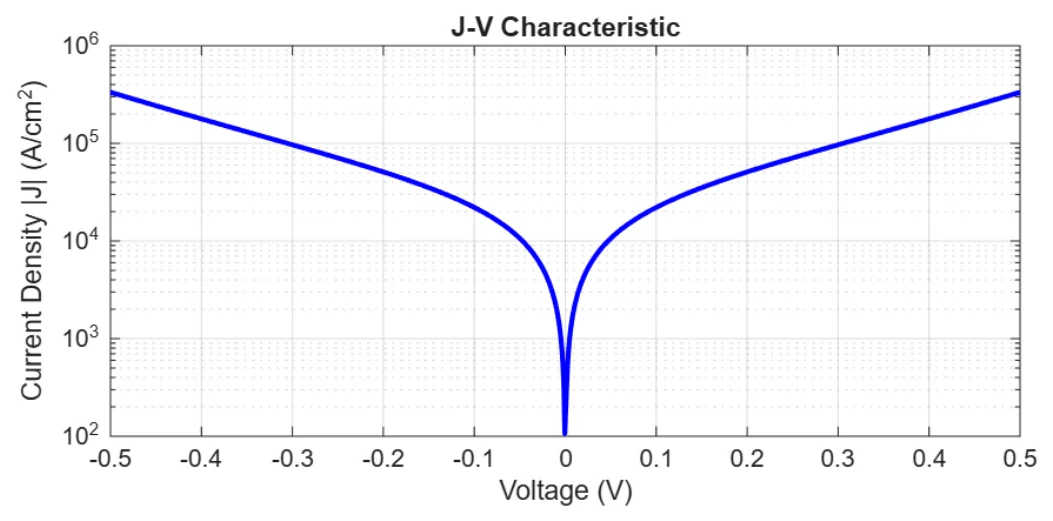
Logarithmic current density–voltage (J–V) characteristic of the NbS_2_–Sc_2_O_3_–Mo_2_C MIM diode.

**Figure 4:**
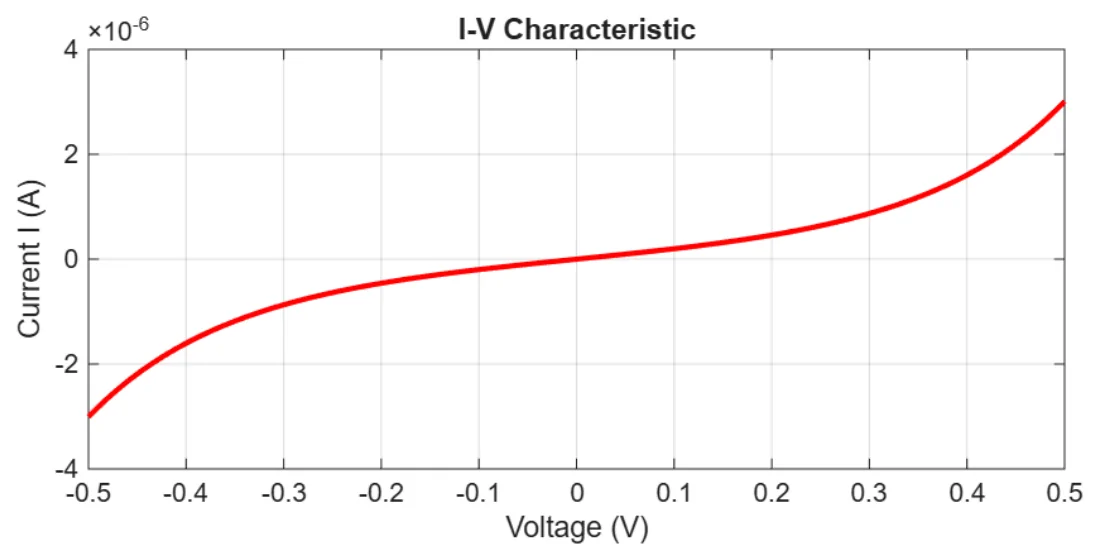
Simulated current–voltage (I–V) characteristic of the NbS_2_–Sc_2_O_3_–Mo_2_C MIM diode.

The NbS_2_–Sc_2_O_3_–Mo_2_C MIM diode was designed by engineering the metal–insulator interfaces to maximize quantum-mechanical tunneling efficiency and rectification performance. The Mo_2_C/Sc_2_O_3_ interface provides a low tunnel-barrier height (0.4 eV), promoting preferential carrier transport in the forward direction, enhancing the tunneling probability, reducing the junction resistance, and improving impedance matching. Conversely, the NbS_2_/Sc_2_O_3_ interface exhibits a higher barrier height (1.70 eV), resulting in a strongly asymmetric potential profile that suppresses the reverse tunneling current, thereby enabling efficient zero-bias rectification. The moderate dielectric constant and favorable electron affinity of Sc_2_O_3_ further reduce parasitic capacitance while improving the RC-limited cutoff frequency. The large work function difference between the NbS_2_ and Mo_2_C electrodes (ΔΨ = 1.30 eV) results in a theoretical asymmetry factor of approximately 2.1 × 10^4^, confirming the strong rectification capability of the proposed device. The electrical characteristics were evaluated in the MATLAB environment using the Simmons tunneling model by sweeping the applied bias voltage from −0.5 to +0.5 V. As shown in Figure 3, the simulated semi-logarithmic J–V characteristic exhibits the exponential dependence expected for direct quantum tunneling, reaching approximately 1.05 × 10^2^ A/cm^2^ at zero bias and 3.34 × 10^5^ A/cm^2^ at ±0.5 V. The corresponding I–V characteristic shown in Figure 4 demonstrates pronounced rectification, with a forward current of 3.0 × 10^−6^ A and a reverse current of only 1.47 × 10^−10^ A. A finite zero-bias current (9.5 × 10^−10^ A) is also observed, confirming the intrinsic tunneling conduction mechanism of asymmetric MIM junctions. These results demonstrate that the NbS_2_–Sc_2_O_3_–Mo_2_C architecture effectively enhances carrier selectivity and rectification performance, making it a promising candidate for LWIR rectenna applications.

**Figure 5:**
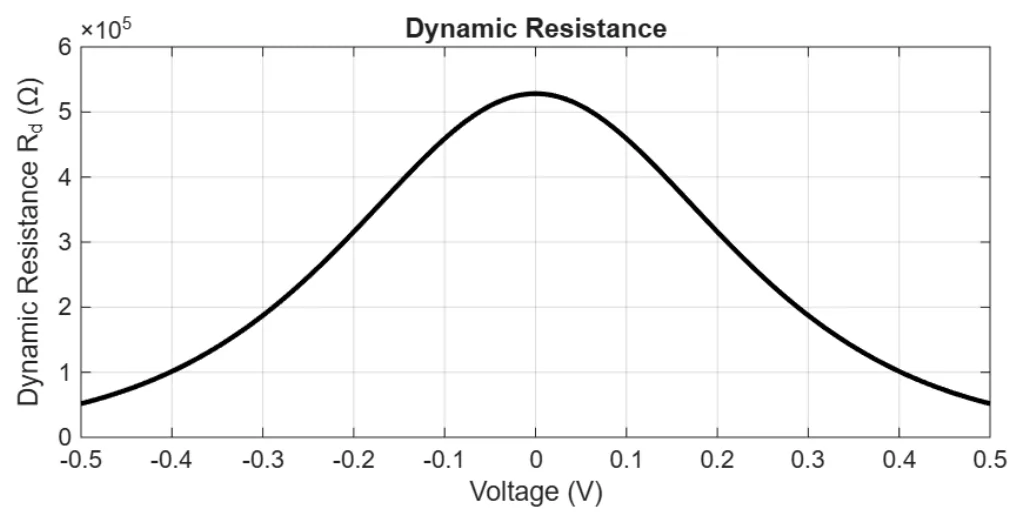
Dynamic resistance versus voltage curve of the NbS_2_–Sc_2_O_3_–Mo_2_C MIM tunnel junction diode.

**Figure 6:**
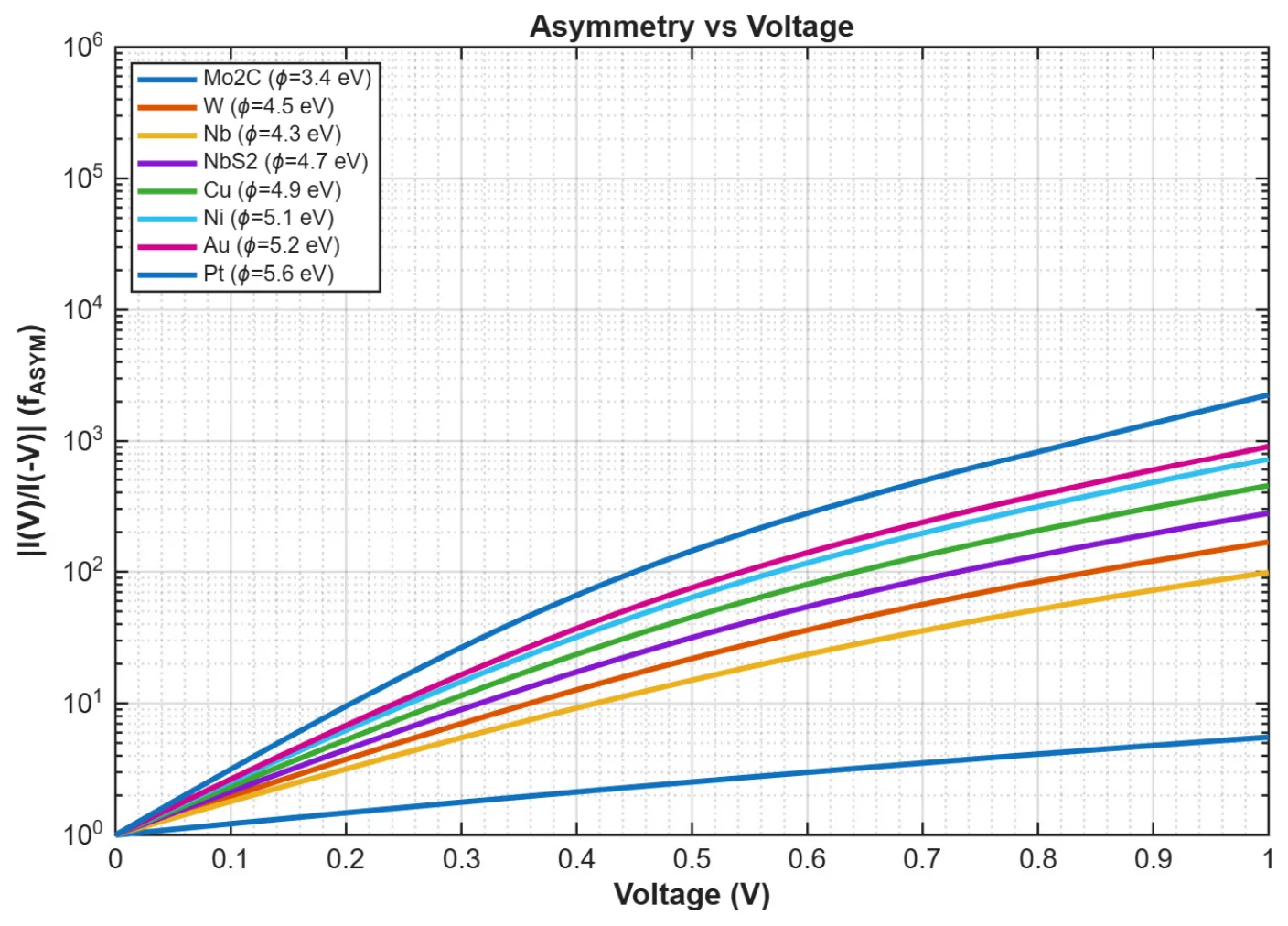
Asymmetry factor as a function of the electrode work function difference ($\Delta \Psi =\Psi_{M1}-\Psi_{M2}$) for various material combinations.

**Figure 7:**
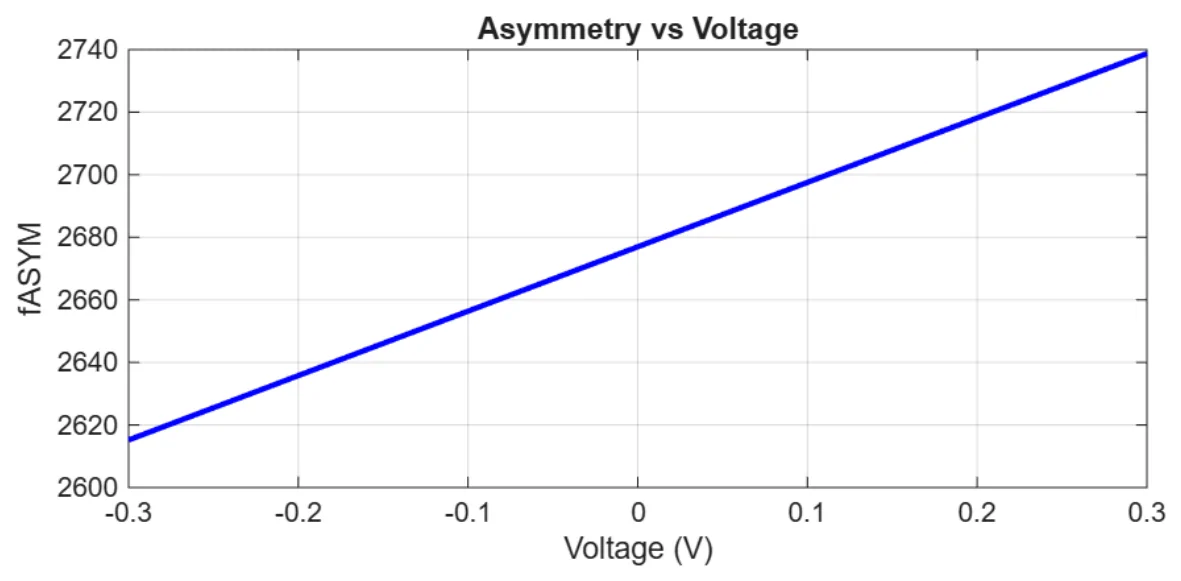
Asymmetry observed at 300 K and plotted as a function of the voltage in the range from −0.3 V to +0.3 V.

Figure 5 shows the bias dependence of the differential resistance (Rd). At zero bias, Rd is approximately 5.3 × 10^5^ Ω and decreases monotonically with increasing applied voltage, reaching approximately 1.9 × 10^5^ Ω at ±0.3 V and 5.2 × 10^4^ Ω at ±0.5 V. This reduction in Rd results from the bias-induced enhancement of the tunneling probability and is consistent with the characteristic transport behavior of asymmetric MIM tunnel junctions. Figure 6 illustrates the dependence of the asymmetry factor (f_ASYM_) on the electrode work function difference (ΔΨ) over the applied voltage range of 0–1 V. Among the investigated material combinations, the NbS_2_–Mo_2_C electrode pair exhibits the highest rectification performance, achieving a theoretical asymmetry factor of approximately 2.1 × 10^4^. These results demonstrate that engineering the electrode work function difference represents an effective strategy for enhancing barrier asymmetry, thereby improving zero-bias rectification, responsivity, and the overall performance of MIM diodes for THz and LWIR energy-harvesting applications. Figure 7 shows an asymmetry factor of 2680, confirming the intrinsic rectification capability of the proposed NbS_2_–Sc_2_O_3_–Mo_2_C MIM diode.

**Figure 8:**
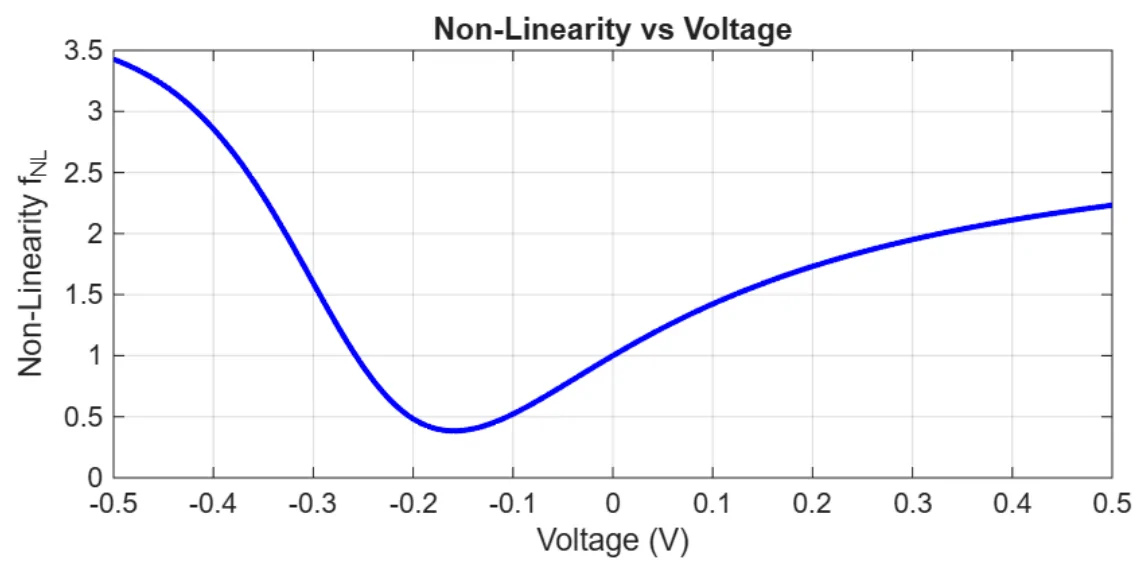
Nonlinearity vs. Voltage (V) of the proposed NbS_2_–Sc_2_O_3_–Mo_2_C MIM diode.

Figure 8 presents the evolution of the nonlinearity factor (f_NL_) with applied voltage. Around zero bias, f_NL_ approaches unity, indicating a pronounced nonlinear response. The response becomes increasingly nonlinear as the bias moves away from zero, reflecting the strong voltage sensitivity of electron tunneling through the ultrathin barrier.

**Figure 9:**
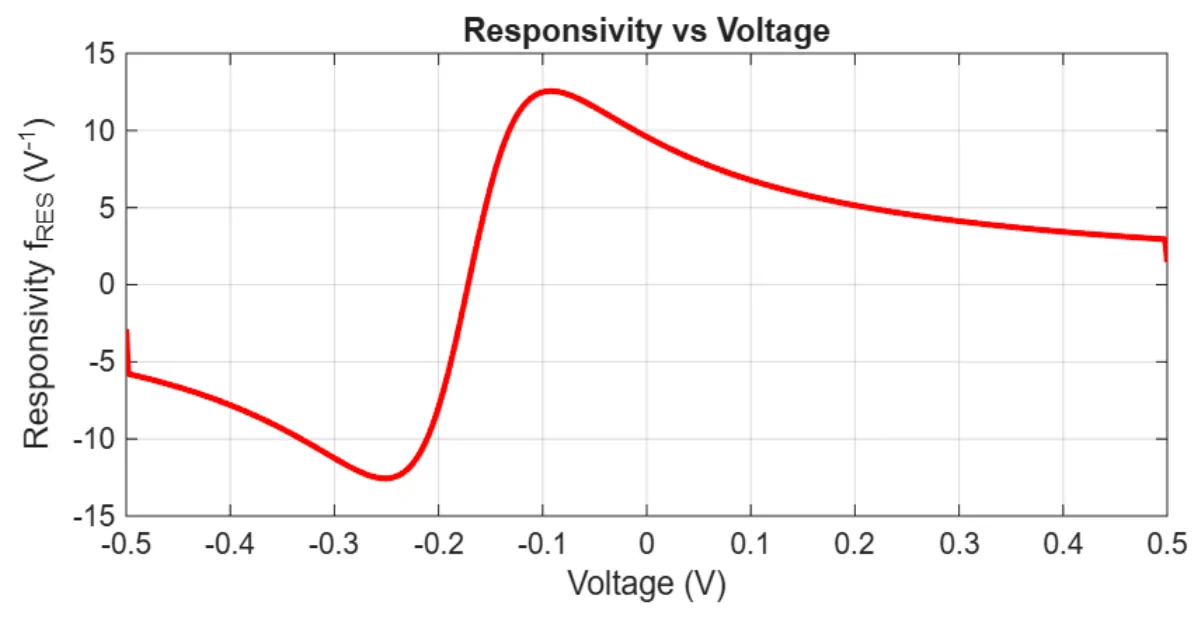
Responsivity versus voltage of the proposed NbS_2_–Sc_2_O_3_–Mo_2_C MIM diode.

Figure 9 demonstrates that the responsivity reaches approximately 10 V^−1^ at zero bias, exceeding the ∼7 V^−1^ [6] value typically regarded as the threshold for efficient self-biased rectification. Furthermore, at a low applied voltage of 0.1 V, the responsivity remains close to 7 V^−1^, indicating excellent low-bias sensitivity and stable rectification performance. This enhanced response is attributed to the optimized asymmetric tunnel barrier, which promotes quantum-mechanical tunneling and generates a highly nonlinear current–voltage characteristic.

### 3.3. VSe_2_/LaOF/VN MIM Diode

**Figure 10:**
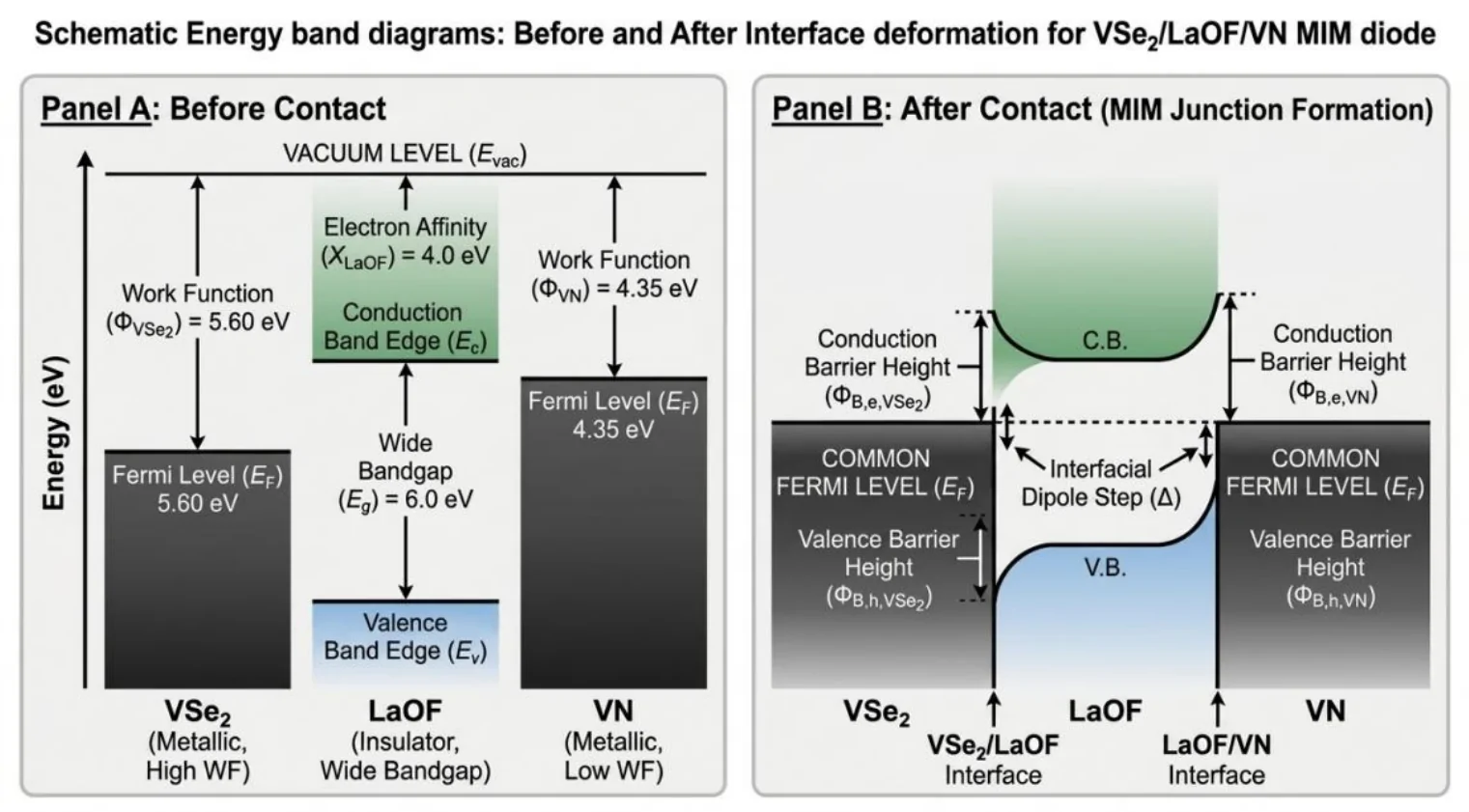
Schematic energy band diagrams of the VSe_2_-LaOF-VN heterostructure. (**A**) Before contact: energy level alignment referenced to a common vacuum level (Evac), based on the work functions of VSe_2_ (5.60 eV) and VN (4.35 eV), and the electron affinity of LaOF (4.00 eV). (**B**) After contact (interface formation): realistic band diagram under thermodynamic equilibrium showing unified Fermi level (EF) alignment, band bending across the LaOF insulating layer, and interface dipole steps (∆) that define the actual electron (ΦB,n) and hole (ΦB,h) barrier heights at both interfaces.

The proposed vanadium diselenide–lanthanum oxyfluoride–vanadium nitride (VSe_2_/LaOF/VN) MIM diode comprises a VSe_2_ top electrode, an ultrathin LaOF tunnel barrier, and a VN bottom electrode. This material combination was selected to establish an asymmetric energy-barrier profile that promotes quantum-mechanical tunneling and rectification at 28.3 THz. VSe_2_ provides a highly conductive electrode with a high density of states near the Fermi level and a suitable work function for efficient carrier injection, while VN offers high electrical conductivity, chemical stability, and a distinct work function that contributes to the required barrier asymmetry. LaOF was selected as the insulating layer owing to its wide bandgap, moderate dielectric permittivity (εr = 13), and suitable electron affinity, enabling effective carrier confinement while maintaining a favorable tunneling barrier. The resulting band alignment and work function contrast enhance electron tunneling and nonlinear transport, thereby improving zero-bias rectification and the key diode figures of merit, including asymmetry, nonlinearity, and responsivity. Figure 10 illustrates the evolution of the energy-band alignment of the VSe_2_/LaOF/VN heterostructure before and after interface formation. Before contact (Figure 10A), the electronic energy levels of the individual components are referenced to a common vacuum level (Evac) according to the Schottky–Mott model. VSe_2_ exhibits a relatively high work function of 5.60 eV, whereas VN serves as the lower-work function electrode, with a work function of 4.35 eV. The LaOF dielectric layer has an electron affinity of 4.00 eV. Under this ideal vacuum-level alignment, the electron barrier heights at the VSe_2_/LaOF and VN/LaOF interfaces are estimated to be 1.60 and 0.35 eV, respectively. Upon interface formation (Figure 10B), charge redistribution occurs across the junction to establish thermodynamic equilibrium, resulting in a common Fermi level (E_F_) throughout the structure. The work function difference of 1.25 eV between the two electrodes drives charge transfer and generates an asymmetric electrostatic potential across the ultrathin LaOF barrier, leading to band bending. In addition, local charge rearrangement and metal-induced gap states (MIGS) may give rise to interfacial dipoles (Δ), causing deviations from the ideal Schottky–Mott barrier heights. Consequently, the actual effective tunneling barriers can differ from the values predicted by the ideal vacuum-level alignment, while their pronounced asymmetry remains responsible for the directional tunneling and rectification behavior of the VSe_2_/LaOF/VN junction.

The main geometrical, material, and electrical parameters are summarized in Table 4 [30,31,32].

The VN/LaOF interface provides a low tunnel-barrier height (0.35 eV), promoting preferential carrier transport in the forward direction, enhancing the tunneling probability, reducing the junction resistance, and improving impedance matching. Conversely, the VSe_2_/LaOF interface exhibits a higher barrier height (1.60 eV), resulting in a strongly asymmetric potential profile that suppresses the reverse tunneling current, thereby enabling efficient zero-bias rectification. The electrical performance of the VSe_2_/LaOF/VN MIM diode was evaluated through MATLAB simulations based on the Simmons tunneling model. The applied bias voltage was swept from −0.5 V to +0.5 V, considering a 2 nm LaOF tunnel barrier and a junction area of 21 × 21 nm (441 nm^2^).

**Figure 11:**
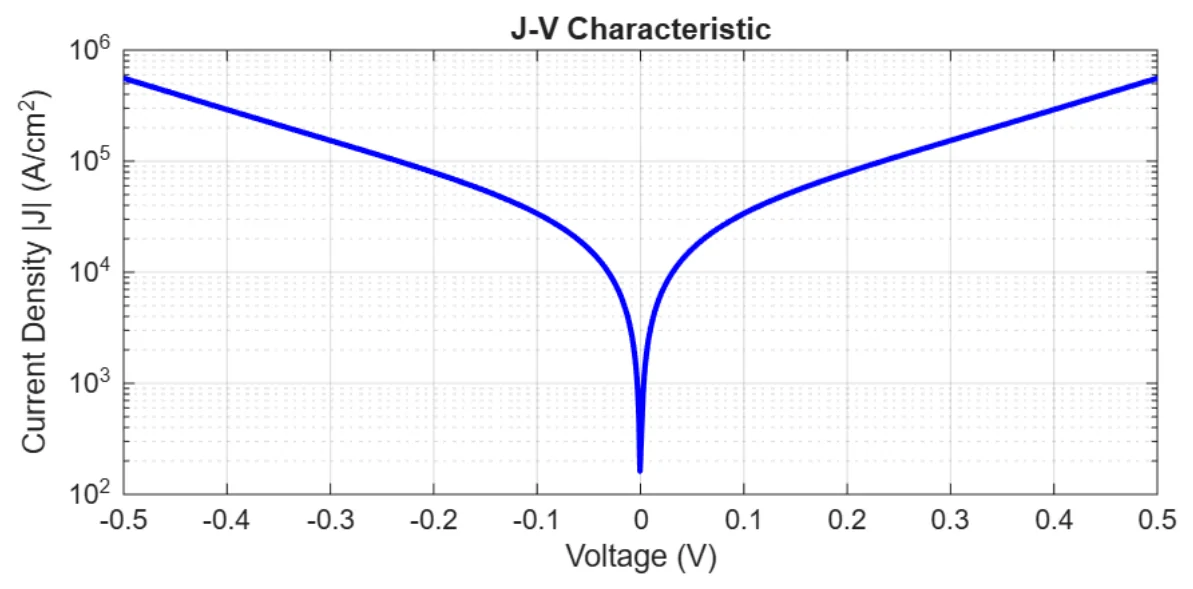
Logarithmic current density–voltage (J–V) characteristic of the VSe_2_-LaOF-VN MIM diode.

**Figure 12:**
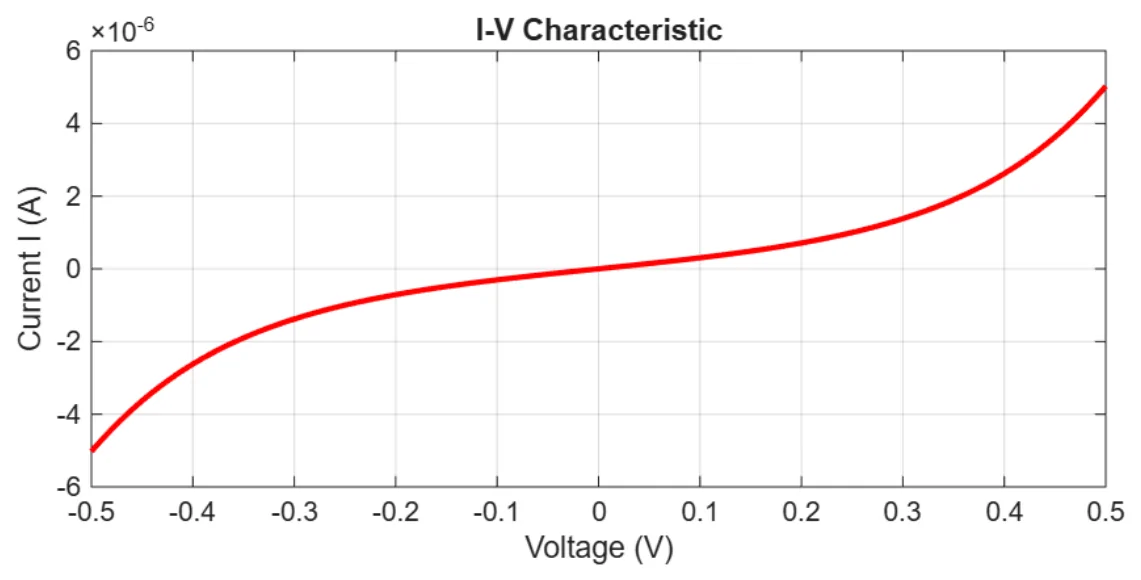
Simulated current–voltage (I–V) characteristic of the VSe_2_-LaOF-VN MIM diode.

As shown in Figure 11, the simulated semi-logarithmic J–V characteristics exhibit the exponential dependence expected for direct quantum tunneling through an ultrathin barrier, with the current density increasing from approximately 1.60 × 10^2^ A/cm^2^ at zero bias to 5.60 × 10^5^ A/cm^2^ at ± 0.5 V. The corresponding I–V characteristics shown in Figure 12 demonstrate pronounced rectification, with a forward current of 5.02 × 10^−6^ A and a reverse current limited to 3.60 × 10^−10^ A. A finite zero-bias current of 1.44 × 10^−9^ A is also observed, confirming the intrinsic tunneling conduction mechanism of the asymmetric MIM junction. These results demonstrate that the VSe_2_/LaOF/VN material combination effectively enhances asymmetric carrier transport and represents a promising platform for high-frequency THz and LWIR rectification applications.

**Figure 13:**
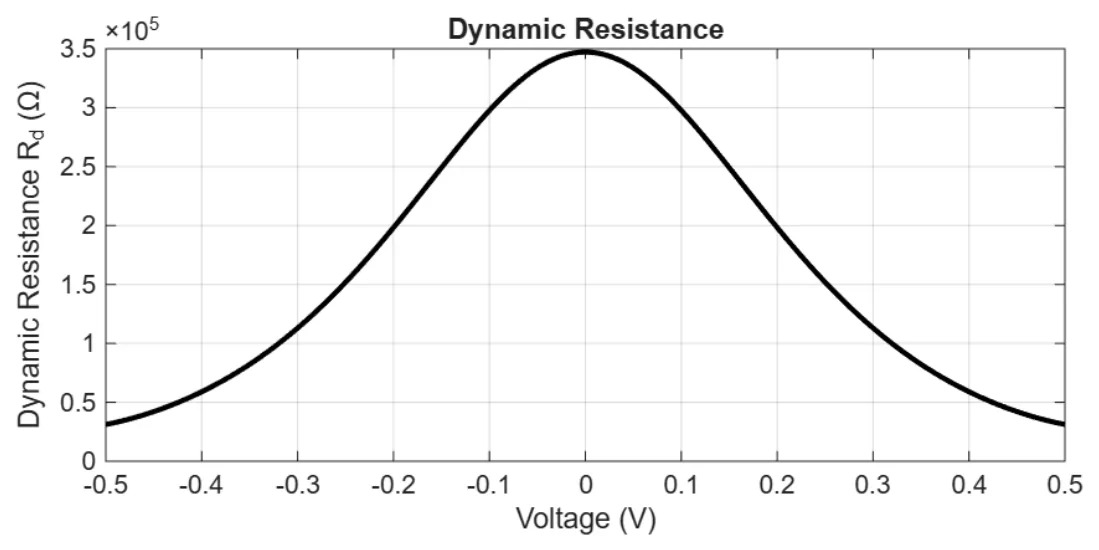
Dynamic resistance versus voltage curve of the VSe_2_-LaOF-VN MIM tunnel junction diode.

Figure 13 shows that the differential resistance decreases from approximately 3.50 × 10^5^ Ω at zero bias to 1.1 × 10^5^ Ω at ±0.3 V and 3.12 × 10^4^ Ω at ±0.5 V, consistent with the exponential increase in tunneling current under applied bias.

**Figure 14:**
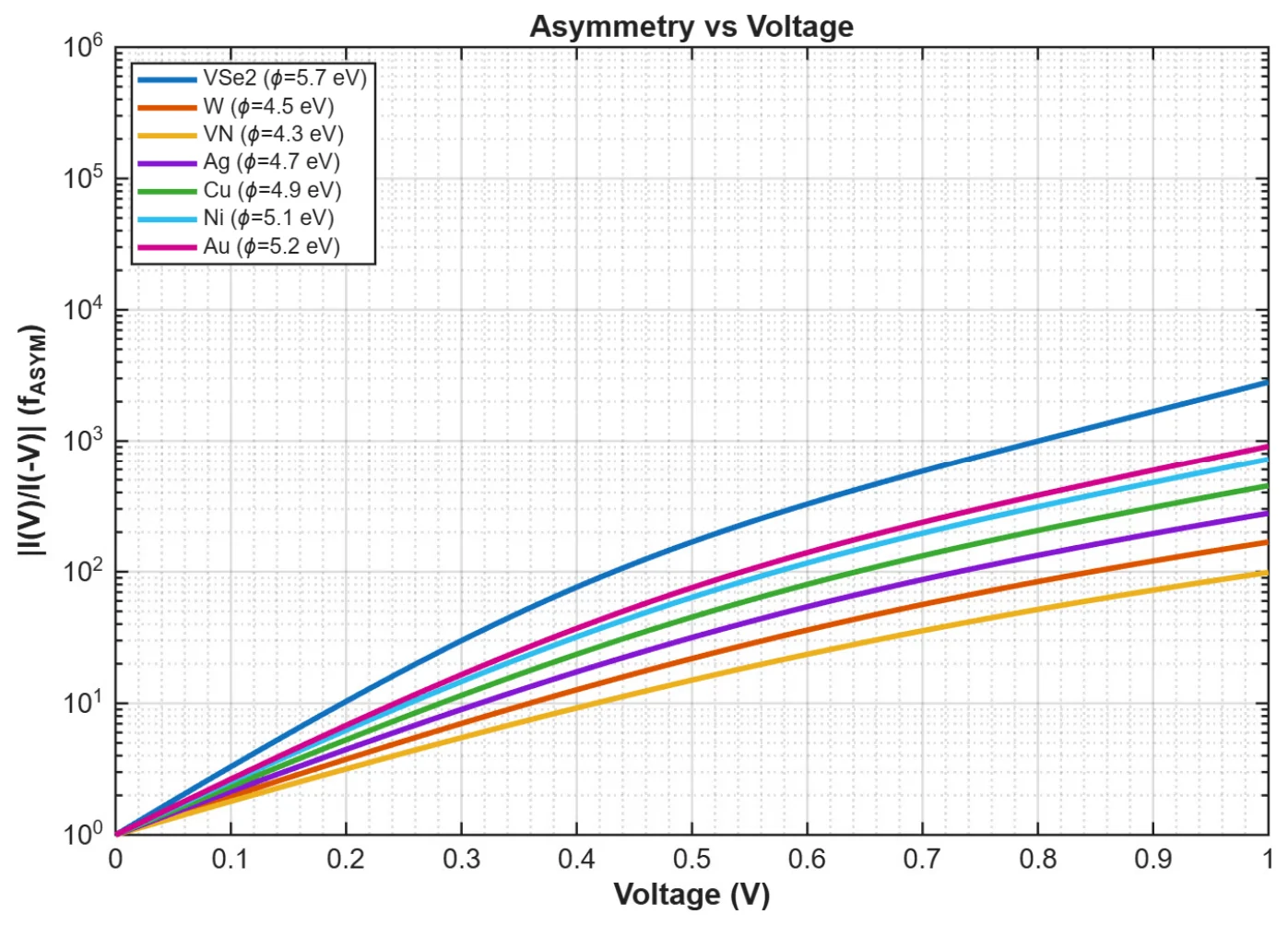
Asymmetry factor as a function of the electrode work function difference ($\Delta \Psi =\Psi_{M1}-\Psi_{M2}$) for various material combinations.

**Figure 15:**
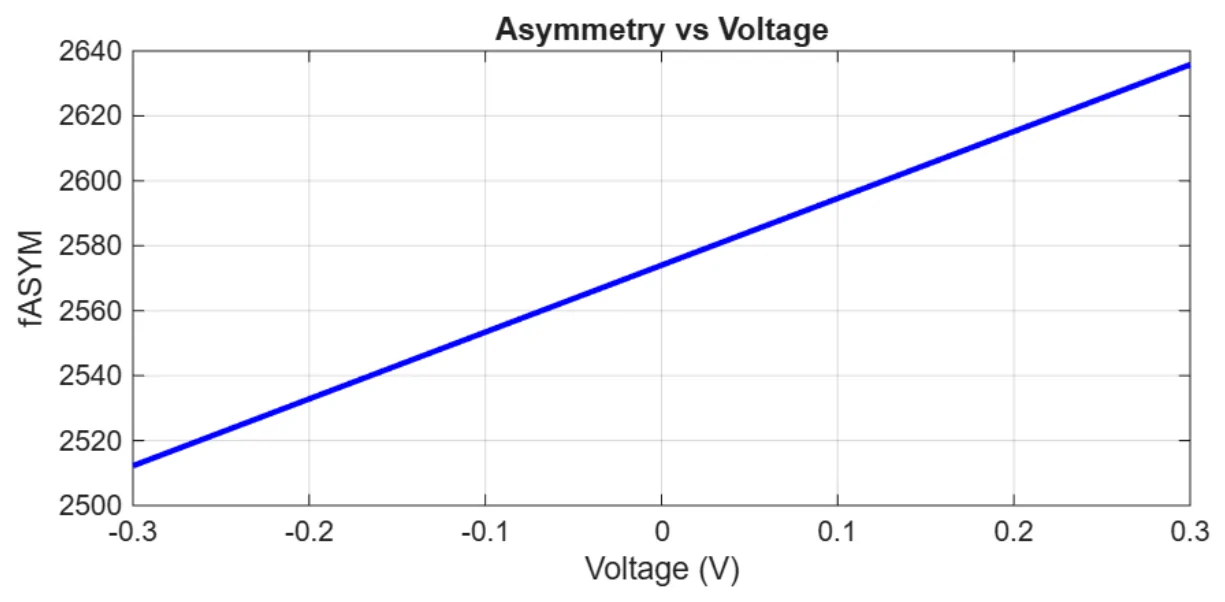
Asymmetry observed at 300 K and plotted as a function of the voltage in the range from −0.3 V to +0.3 V.

Figure 14 presents the asymmetry factor (f_ASYM_) as a function of the electrode work function difference (ΔΨ) over the applied bias voltage range of 0–1 V. Among the investigated material combinations, the VSe_2_–VN electrode pair exhibits the highest rectification performance, achieving an asymmetry factor of approximately 1.4 × 10^4^. Figure 15 shows an asymmetry factor of 2580, confirming the intrinsic rectification capability of the VSe_2_-LaOF-VN MIM diode.

**Figure 16:**
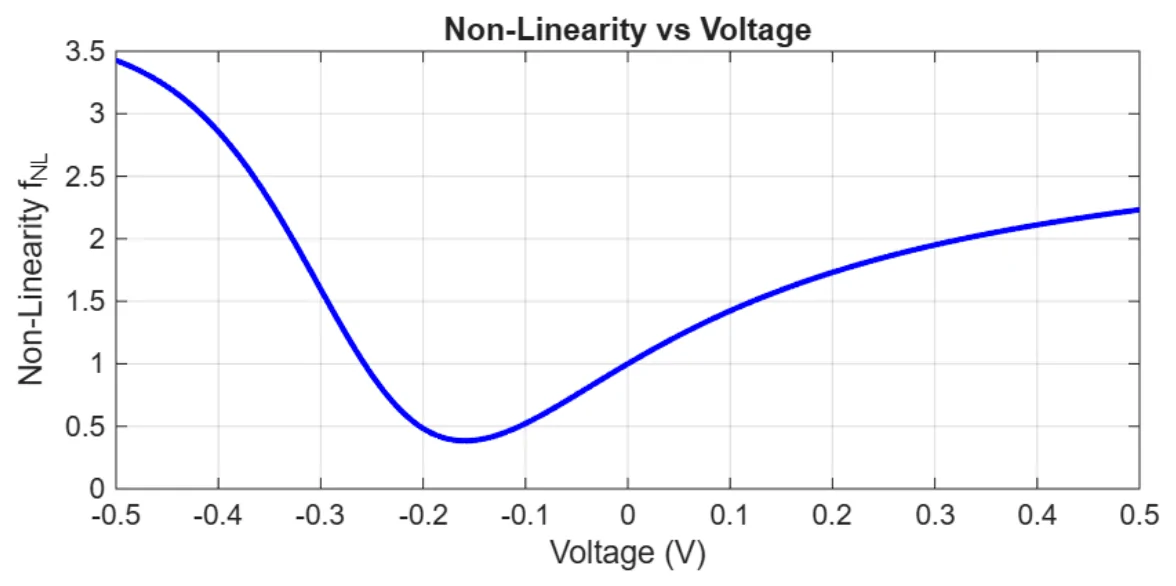
Nonlinearity vs. Voltage (V) of the proposed VSe_2_-LaOF-VN MIM diode.

Figure 16 shows the voltage dependence of the nonlinearity factor (f_NL_). Near zero bias, f_NL_ approaches unity, indicating the nonlinear transport response required for efficient rectification. As the bias increases further, f_NL_ exhibits a progressively stronger nonlinear dependence, reflecting the exponential sensitivity of the tunneling probability to the effective barrier height and width. This voltage-dependent behavior is characteristic of quantum-mechanical transport through ultrathin MIM junctions.

**Figure 17:**
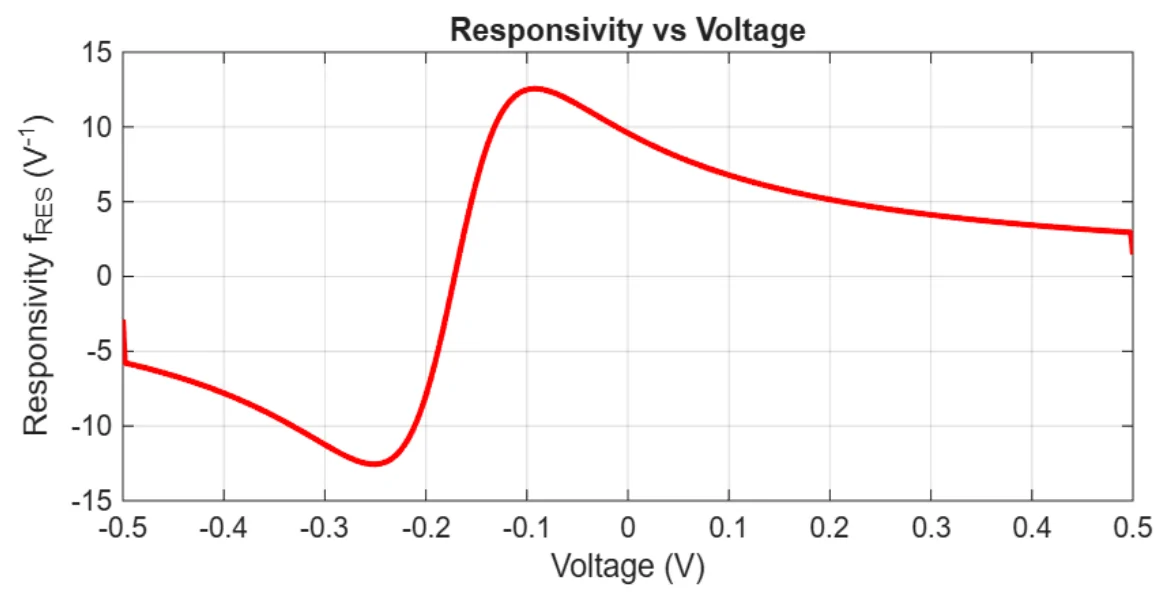
Responsivity versus voltage of the proposed VSe_2_-LaOF-VN MIM diode.

Figure 17 demonstrates that the responsivity reaches approximately 10 V^−1^ at zero bias, exceeding the commonly reported threshold of 7 V^−1^ required for efficient self-biased rectification. Even at a low applied voltage of 0.1 V, the responsivity remains close to 7 V^−1^, indicating excellent low-bias sensitivity and stable rectification performance. This enhanced response is attributed to the optimized tunnel barrier asymmetry, increased electron tunneling probability, and pronounced nonlinear transport behavior of the proposed MIM junction.

### 3.4. TaS_2_/LaOBr/V MIM Diode

**Figure 18:**
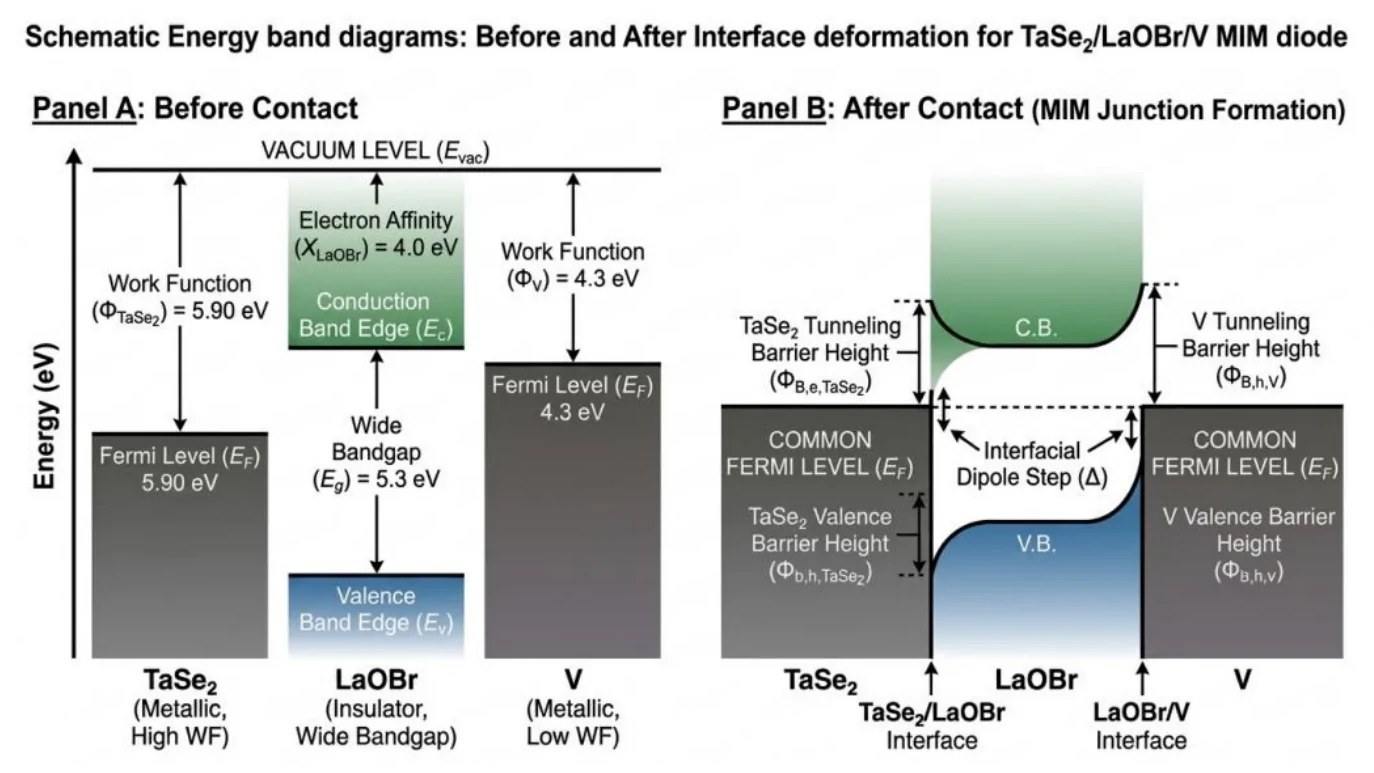
Schematic energy band alignment of the TaSe_2_/LaOBr/V MIM diode. (**A**) Before contact: isolated energy levels aligned to the vacuum level (Evac). (**B**) After contact: equilibrium energy band diagram showing common Fermi level (EF) alignment, band bending across the LaOBr dielectric, and interfacial dipole steps (Δ) defining the realistic tunneling barrier heights.

The proposed tantalum disulfide–lanthanum oxybromide–vanadium (TaS_2_/LaOBr/V) MIM diode comprises a TaS_2_ top electrode, an ultrathin LaOBr tunnel barrier, and a V bottom electrode. This material combination was selected to promote efficient quantum-mechanical tunneling and rectification at the target frequency of 28.3 THz. TaS_2_ was chosen as the top electrode owing to its high electrical conductivity, chemical stability, and suitable work function, which favor efficient carrier injection. LaOBr serves as the insulating layer, combining a wide bandgap, moderate dielectric permittivity (εr = 9), and appropriate electron affinity to provide a well-defined tunnel barrier while limiting leakage currents. The V bottom electrode, with its lower work function and high electrical conductivity, introduces a pronounced barrier asymmetry with respect to TaS_2_, favoring preferential electron transport and suppressing reverse tunneling. The resulting asymmetric energy-band profile, together with the ultrathin LaOBr barrier, enhances the tunneling probability and nonlinear current response, thereby promoting efficient zero-bias rectification at 28.3 THz. As illustrated in Figure 18, the energy-band evolution of the TaSe_2_/LaOBr/V MIM diode was analyzed before and after interface formation to overcome the limitations of simple vacuum-level alignment in describing realistic interfacial electronic structures. Before contact (Figure 18A), the energy levels of the individual materials are referenced to a common vacuum level (Evac), using the work functions of TaSe_2_ (ΦTaSe_2_ = 5.90 eV) and V (ΦV = 4.30 eV), together with the electron affinity (χLaOBr = 4.0 eV) and bandgap (Eg = 5.3 eV) of LaOBr. Upon interface formation (Figure 18B), charge redistribution occurs to establish thermodynamic equilibrium, resulting in a common Fermi level (E_F_) across the junction. The substantial work function difference (ΔΦ = 1.60 eV) generates an asymmetric electrostatic potential and pronounced band bending across the ultrathin LaOBr tunneling barrier. Interfacial charge rearrangement may additionally give rise to dipole-induced potential steps (Δ), modifying the effective barrier heights relative to the ideal Schottky–Mott prediction. The resulting asymmetric electron tunneling barriers, approximately 1.90 eV at the TaSe_2_/LaOBr interface and 0.30 eV at the V/LaOBr interface, are therefore expected to play a central role in determining the directional tunneling transport and rectification performance of the MIM diode.

The main geometrical, material, and electrical parameters are summarized in Table 5 [33,34,35].

**Figure 19:**
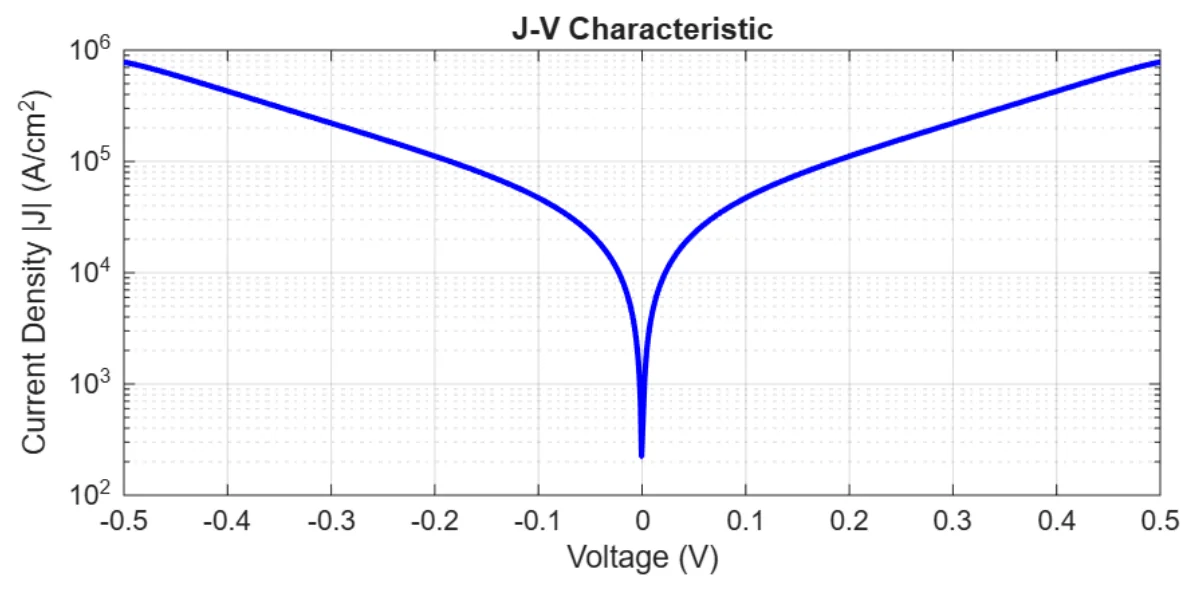
Logarithmic current density–voltage (J–V) characteristic of the TaS_2_/LaOBr/V MIM diode.

**Figure 20:**
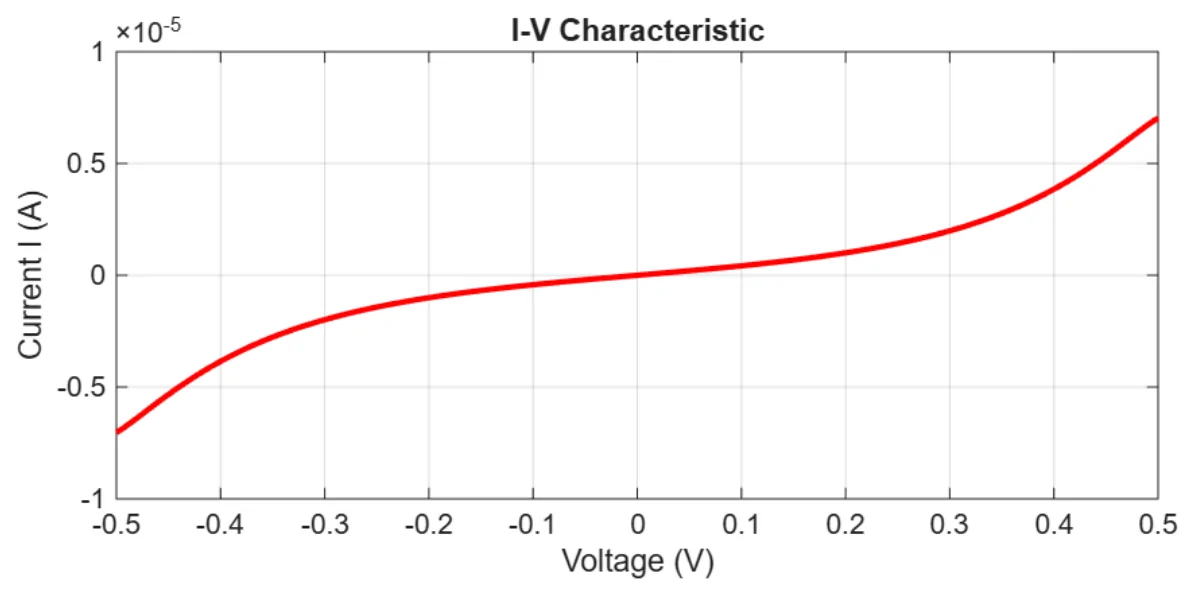
Simulated current–voltage (I–V) characteristic of the TaS_2_/LaOBr/V MIM diode.

The V/LaOBr interface exhibits a low electron tunneling barrier height of 0.30 eV, which enhances the tunneling probability, facilitates carrier transport, and reduces junction resistance. LaOBr provides a favorable combination of electron affinity and a moderate dielectric constant (εr ≈ 9), enabling efficient nonlinear rectification while minimizing parasitic capacitance. Conversely, the TaS_2_/LaOBr interface forms a higher barrier height of 1.90 eV, resulting in a strongly asymmetric potential profile that promotes directional carrier transport and efficient zero-bias rectification. The electrical characteristics of the TaS_2_/LaOBr/V MIM diode were investigated using MATLAB simulations based on the Simmons tunneling formalism. The applied bias voltage was swept from −0.5 V to +0.5 V to evaluate the resulting J–V and I–V characteristics and assess the rectification performance of the asymmetric tunneling junction. As shown in Figure 19, the simulated semi-logarithmic J–V characteristic exhibits the exponential dependence expected for direct quantum-mechanical tunneling through an ultrathin barrier. For a junction area of 22 × 22 nm (490 nm^2^), the current density reaches approximately 2.2 × 10^2^ A/cm^2^ at zero bias and increases to 7.8 × 10^5^ A/cm^2^ at ±0.5 V. The corresponding I–V characteristic shown in Figure 20 confirms the strong rectification behavior induced by the asymmetric tunneling barrier. Under forward bias, the current reaches approximately 7 × 10^−6^ A, whereas the reverse current remains limited to 3.5 × 10^−11^ A, demonstrating pronounced transport asymmetry. A finite zero-bias current of approximately 2 × 10^−9^ A is also observed, consistent with the intrinsic tunneling transport mechanism of asymmetric MIM junctions. These results confirm the effectiveness of the TaS_2_/LaOBr/V material system in promoting directional carrier transport and achieving efficient rectification through optimized tunnel barrier asymmetry [33,34,35].

**Figure 21:**
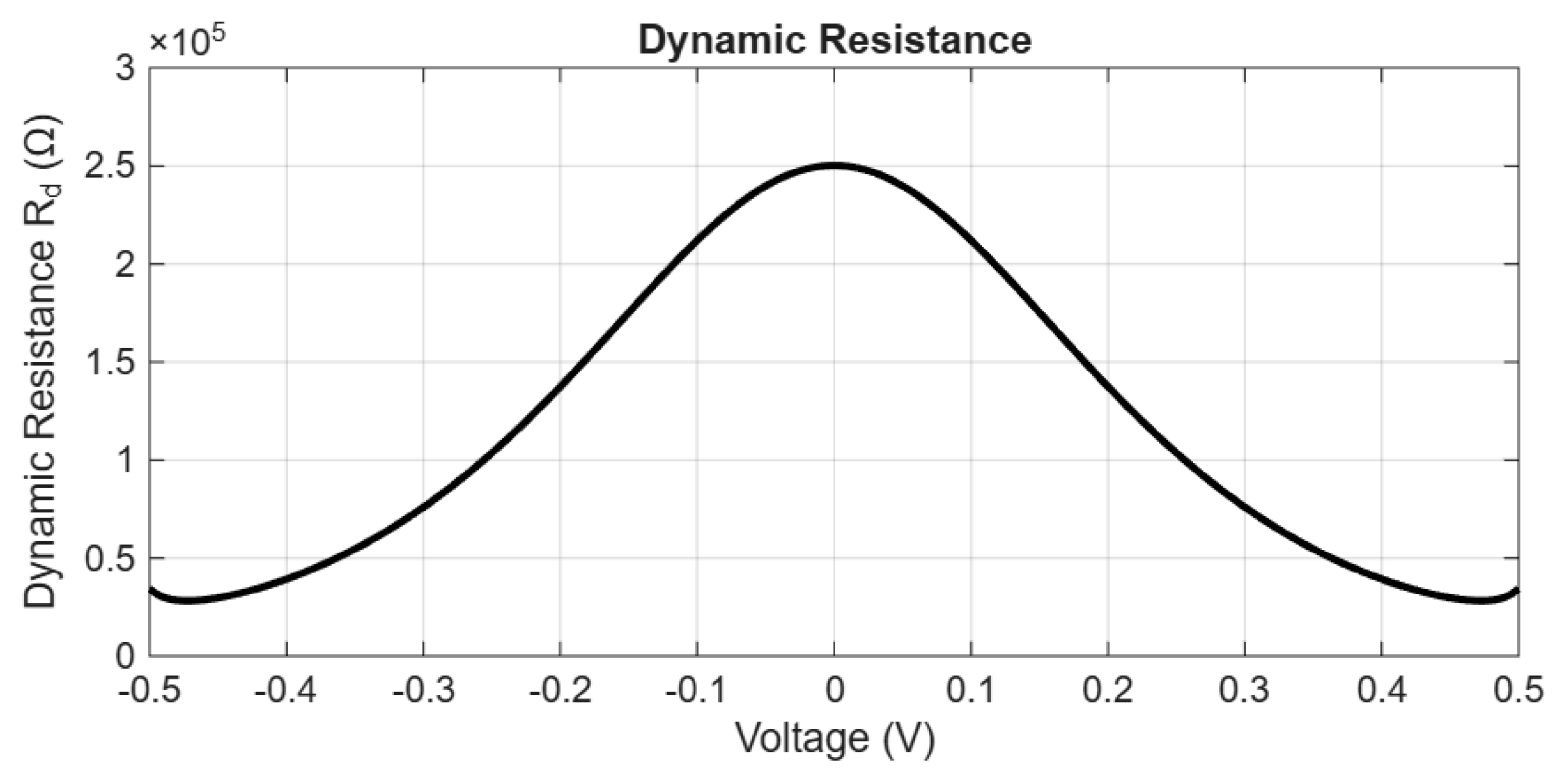
Dynamic resistance versus voltage curve of the TaS_2_/LaOBr/V MIM tunnel junction diode.

Figure 21 shows the bias dependence of the differential resistance (Rd). At zero bias, Rd is approximately 2.5 × 10^5^ Ω and decreases monotonically with increasing applied voltage, reaching approximately 7.5 × 10^4^ Ω at ±0.3 V and 3.4 × 10^4^ Ω at ±0.5 V. This reduction in Rd results from the bias-induced enhancement of the tunneling probability and is consistent with the characteristic transport behavior of asymmetric MIM tunnel junctions.

**Figure 22:**
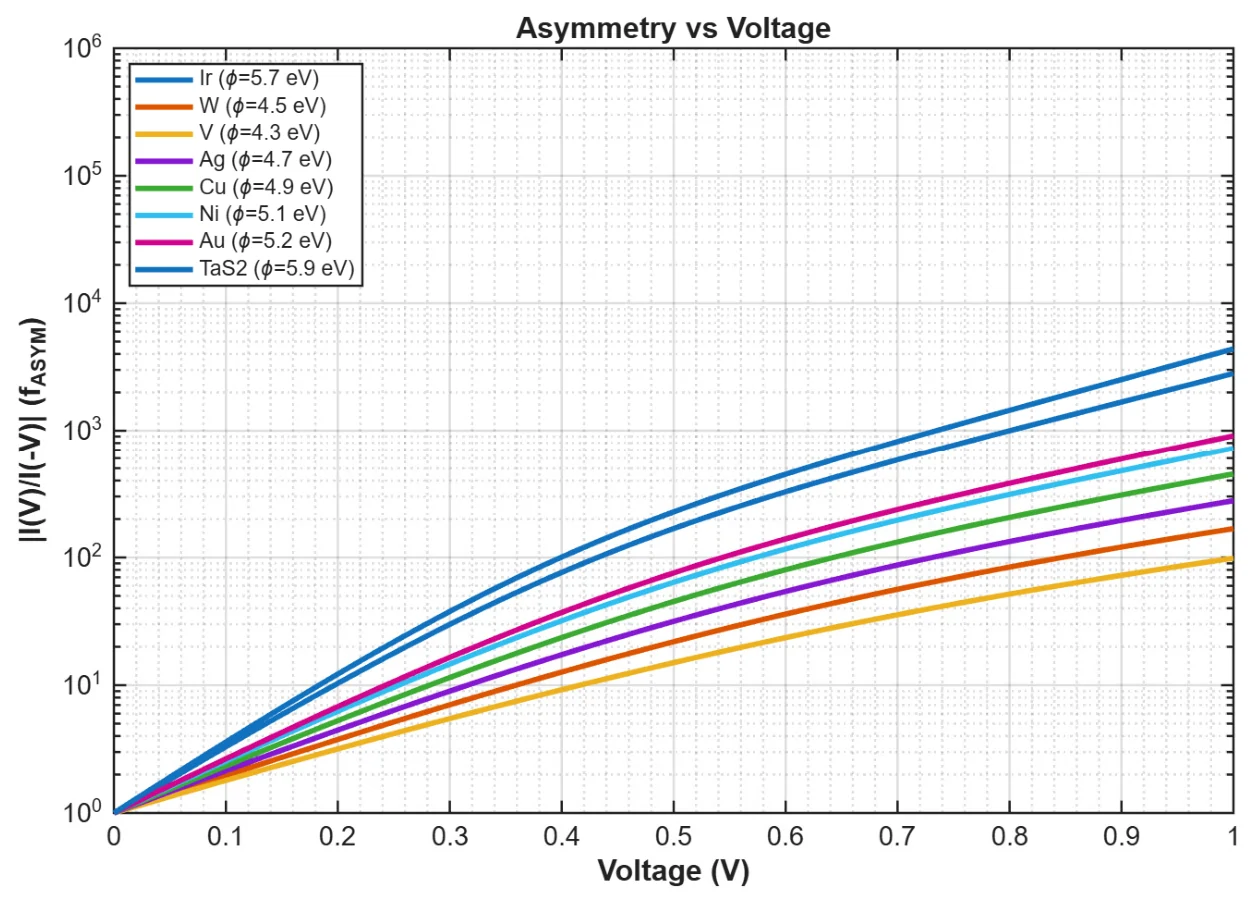
Asymmetry factor as a functionof the electrode work function difference ($\Delta \Psi =\Psi_{M1}-\Psi_{M2}$) for various material combinations.

**Figure 23:**
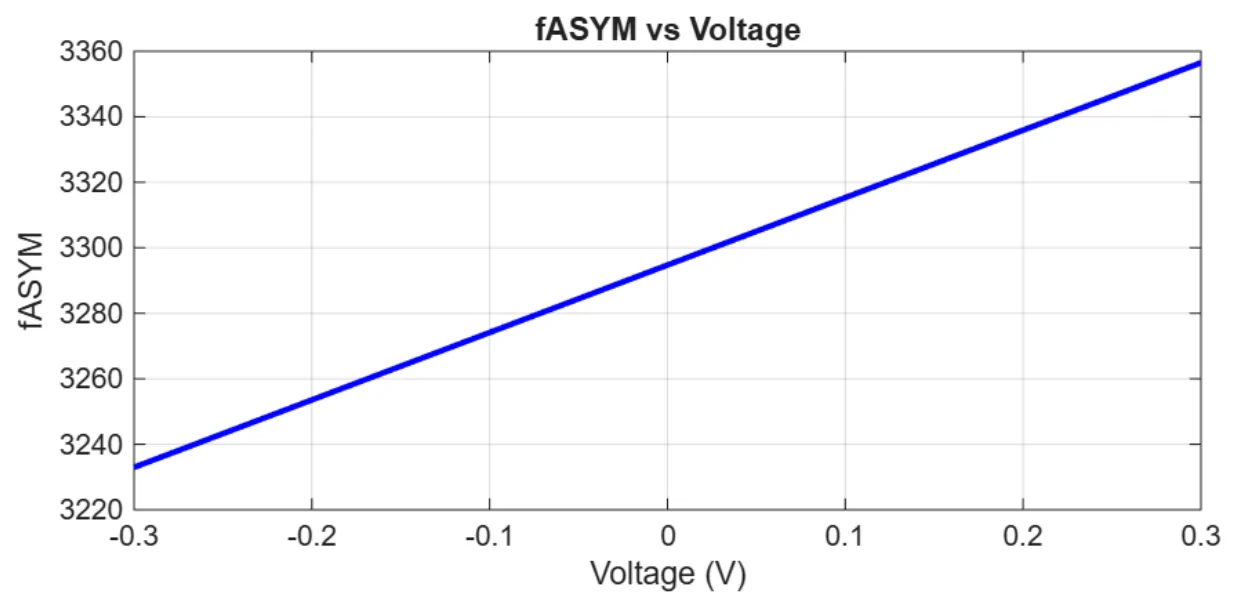
Asymmetry observed at 300 K and plotted as a function of the voltage in the range from −0.3 V to +0.3 V.

Figure 22 illustrates the variation in the asymmetry factor (f_ASYM_) as a function of the electrode work function difference (ΔΨ) over the applied bias voltage range of 0–1 V. For the proposed TaS_2_/LaOBr/V MIM diode, the electrode work function difference (ΔΨ = 1.6 eV) generates a highly asymmetric tunneling barrier, resulting in an asymmetry factor of approximately f_ASYM_ ≈ 2 × 10^5^. This pronounced barrier asymmetry enhances forward electron tunneling while suppressing reverse current flow, thereby confirming the effectiveness of work function engineering in improving the rectification performance of MIM diodes. Figure 23 shows an asymmetry factor of 3300, confirming the intrinsic rectification capability of the TaS_2_/LaOBr/V MIM diode.

**Figure 24:**
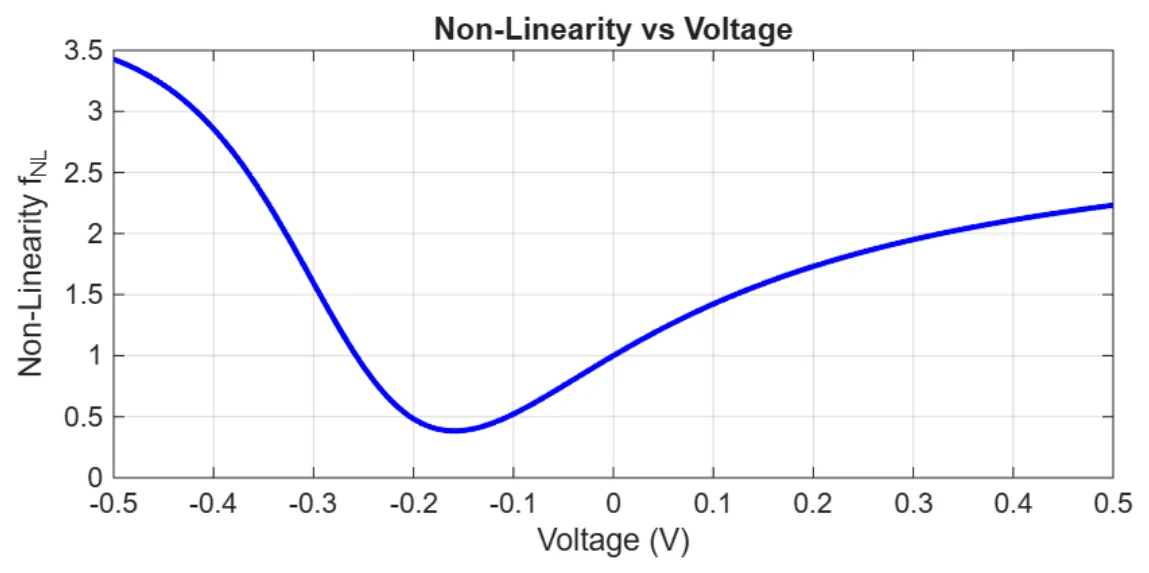
Non-linearity vs. Voltage (V) of the proposed TaS_2_/LaOBr/V MIM diode.

Figure 24 illustrates the voltage dependence of the nonlinearity factor (f_NL_). Near zero bias, f_NL_ approaches unity, indicating the nonlinear transport response required for efficient rectification. As the bias increases further, f_NL_ exhibits a progressively stronger nonlinear dependence, reflecting the exponential sensitivity of the tunneling probability to the effective barrier height and width. This voltage-dependent behavior is characteristic of quantum-mechanical transport through ultrathin MIM junctions.

**Figure 25:**
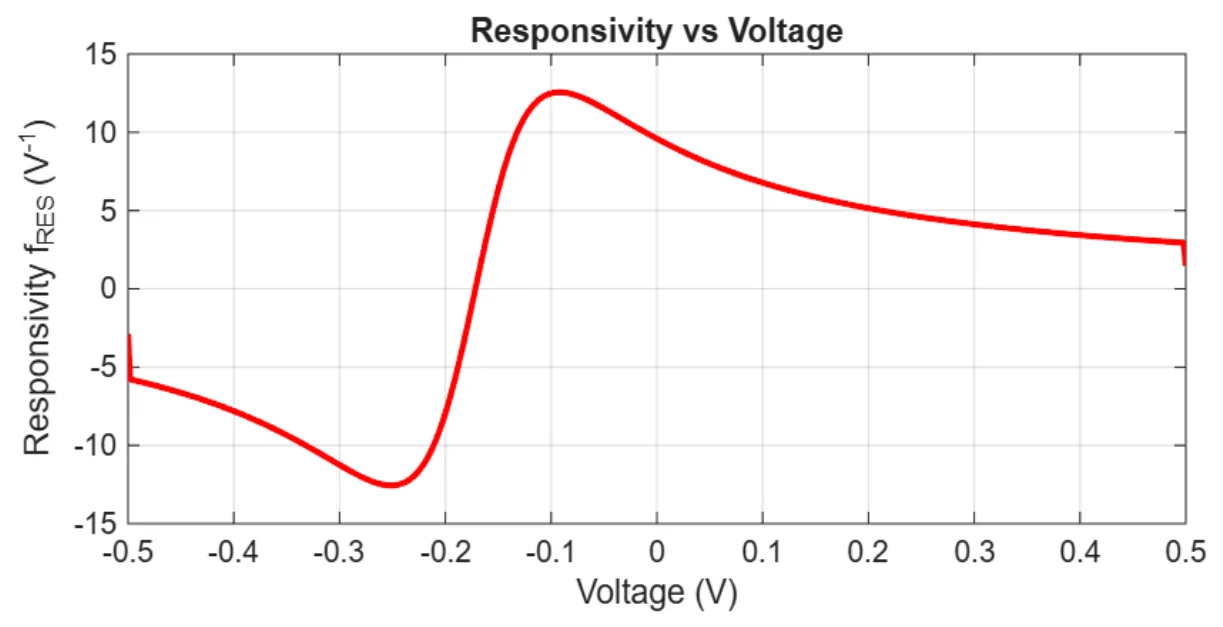
Responsivity versus voltage of the proposed TaS_2_/LaOBr/V MIM diode.

Figure 25 shows that the responsivity reaches approximately 10 V^−1^ at zero bias, exceeding the ∼7 V^−1^ benchmark generally considered necessary for efficient self-biased rectification. Even at an applied bias voltage of 0.1 V, the responsivity remains close to 7 V^−1^, demonstrating sustained high sensitivity in the low-bias regime. This performance is attributed to the optimized tunnel barrier asymmetry and enhanced quantum-mechanical tunneling, which together promote efficient carrier transport and a pronounced nonlinear current–voltage response.

## 4. Comparative Analysis of the Proposed MIM Diodes Using FOM and Electrical Parameters

The three proposed MIM diode architectures—NbS_2_/Sc_2_O_3_/Mo_2_C, VSe_2_/LaOF/VN, and TaS_2_/LaOBr/V—were systematically evaluated using FOM parameters. The analysis aimed to identify the material combination offering the optimal balance between tunneling efficiency, nonlinear rectification, and zero-bias operation for high-frequency LWIR energy-harvesting applications. Variations in tunnel barrier height, dielectric properties, and electrode work function difference produce distinct quantum-mechanical tunneling and rectification characteristics for each device. The NbS_2_/Sc_2_O_3_/Mo_2_C diode exhibits strong rectification with high asymmetry and moderate tunneling efficiency, whereas the VSe_2_/LaOF/VN structure provides an improved balance between responsivity and nonlinear transport behavior. Among the investigated architectures, the TaS_2_/LaOBr/V diode exhibits the best overall performance, combining a low tunnel-barrier height, enhanced tunneling efficiency, high asymmetry, and excellent zero-bias rectification, making it the most promising candidate for LWIR rectenna applications. A comprehensive comparison of the principal electrical parameters and rectification figures of merit for the three proposed MIM diode architectures is presented in Table 6.

### Technical Feasibility and Processing Considerations for the TaS_2_/LaOBr/V Structure

The asymmetric TaS_2_/LaOBr/V MIM diode is technically feasible from a materials-integration and device-fabrication perspective, provided that appropriate thin-film deposition and interface-control strategies are adopted. The fabrication procedure should be designed to ensure precise control of the ultrathin LaOBr tunnel barrier and to minimize contamination and uncontrolled oxidation at the interfaces. Since tunneling current depends exponentially on barrier thickness, precise control of the LaOBr thickness is essential. Atomic layer deposition (ALD) could provide accurate thickness control and high film uniformity. Particular attention should be paid to the TaS_2_/LaOBr interface, as well as to the LaOBr/V interface. The TaS_2_ and V electrodes can, in principle, be integrated using established thin-film and micro/nanofabrication approaches. Contamination, defects, and interfacial reactions may modify the effective tunneling barrier and introduce additional electronic states. In particular, oxidation of the V electrode could form an uncontrolled oxide layer in series with the LaOBr barrier, thereby altering the MIM tunneling characteristics. Therefore, controlled deposition conditions, appropriate surface cleaning, and minimized air exposure between fabrication steps are essential to preserve the designed interface properties. The proposed TaS_2_/LaOBr/V MIM diode is then integrated with the gold receiving antenna through a sequential thin-film deposition and nanofabrication process. First, the gold antenna is fabricated on a suitable substrate, optionally incorporating a dielectric matching layer, by physical vapor deposition followed by lithographic patterning to define the two antenna arms and the nanoscale feed gap. The TaS_2_/LaOBr/V junction is then formed within the antenna feed gap. The TaS_2_ electrode is electrically connected to one antenna arm, while the V electrode is connected to the opposite arm, ensuring that the voltage induced by the antenna is directly applied across the asymmetric MIM junction. TaS_2_ can be deposited or transferred onto the pre-patterned structure and subsequently patterned to define the bottom electrode. After appropriate surface cleaning, the ultrathin LaOBr layer is deposited to form the tunnel barrier, followed by deposition and patterning of the V top electrode to define the active junction area. High-resolution lithography and alignment markers can be employed to ensure accurate nanoscale alignment.

The gold antenna acts as the electromagnetic receiving element and couples the incident 28.3 THz radiation to the TaS_2_/LaOBr/V junction, while the asymmetric tunnel barrier provides the nonlinear current–voltage response required for rectification. Therefore, practical realization will primarily require optimization of the tunnel-barrier uniformity, interface quality, contact resistance, and nanoscale alignment.

## 5. Conclusions and Future Work

A theoretical and numerical investigation based on a quantum-mechanical tunneling model was performed to assess the electrical performance of three planar MIM diode architectures based on the NbS_2_/Sc_2_O_3_/Mo_2_C, VSe_2_/LaOF/VN, and TaS_2_/LaOBr/V material systems. At the target operating frequency of 28.3 THz, the proposed architectures were evaluated from their simulated J–V and I–V characteristics at 300 K, from which the corresponding FOMs were extracted. The results demonstrate that device performance is primarily determined by the tunnel-barrier height and asymmetry, dielectric properties, and electrode work functions. The NbS_2_/Sc_2_O_3_/Mo_2_C diode exhibited the highest zero-bias resistance, providing effective current suppression but comparatively lower tunneling efficiency due to its relatively high effective barrier height. The VSe_2_/LaOF/VN structure exhibited enhanced tunneling transport, resulting in higher current density, improved nonlinearity, and increased responsivity while maintaining favorable rectification characteristics. Among the investigated architectures, the TaS_2_/LaOBr/V diode delivered the best overall performance. Its optimized asymmetric tunnel-barrier profile and low metal–insulator barrier height resulted in the highest asymmetry factor (>2.5 × 10^5^), together with a high zero-bias responsivity (≈10 V^−1^), pronounced nonlinear behavior, and reduced junction resistance. These characteristics promote efficient electron tunneling while preserving strong rectification, making TaS_2_/LaOBr/V the most promising architecture for LWIR rectenna applications. Overall, the results indicate that maximizing the performance of MIM tunneling diodes requires the simultaneous optimization of the electrode work functions, tunnel-barrier height and asymmetry, dielectric permittivity, and barrier thickness. Although the proposed architectures exhibit significantly improved electrical FOMs compared with conventional MIM diodes reported in Table 2, diode resistance and antenna–diode impedance mismatch remain important challenges for practical energy harvesting. Further optimization of electromagnetic coupling, impedance matching, and power-transfer efficiency is therefore required to fully exploit the potential of THz LWIR rectennas.

## Figures and Tables

**Table 1:** Electrical performance analysis of the MIM diode and corresponding FOM [6].

FOM	Definition	Key Notes/Trade-Offs
Zero-Bias Dynamic Resistance ($R_{0}$) Ω $R_{0}={\left(\frac{\mathrm{dI}}{\mathrm{dV}}\right)}_{V=0}^{-1}$	Small-signal resistance at zero bias, characterizing the differential V–I response near the operating point.	Determines the antenna–rectifier impedance matching by optimizing the barrier height and thickness.
Asymmetry ($f_{\mathrm{ASYM}}$) $f_{\mathrm{asym}}(V)=\frac{I_{\mathrm{ON}}}{I_{\mathrm{OFF}}}=\left|\frac{I_{F}(V)}{I_{R}(V)}\right|$	Quantifies the imbalance between the forward IF and reverse currents IR, reflecting the diode’s rectification capability.	Values greater than unity indicate enhanced rectification, which can be tuned by controlling the work function difference between the metal electrodes.
Nonlinearity ($f_{\mathrm{NL}}$) $f_{\mathrm{NL}}(V)=\frac{\frac{\mathrm{dI}}{\mathrm{dV}}(V)}{\frac{I(V)}{V}}$	Quantifies the degree of nonlinearity of the diode’s I–V characteristic, reflecting its deviation from linear conduction.	Values greater than 3 indicate strong rectification, governed by the barrier geometry, thickness, and degree of asymmetry.
Responsivity ($f_{\mathrm{RES}}$) V^−1^ $f_{\mathrm{RES}}(V)=\frac{\frac{d^{2}I}{d^{2}V}(V)}{\frac{\mathrm{dI}}{\mathrm{dV}}(V)}$	Quantifies the efficiency of converting incident electromagnetic energy into electrical energy.	Values greater than 7 indicate efficient zero-bias rectification, enhanced by barrier asymmetry and optimized barrier thickness.
TOV	Minimum bias voltage required to achieve substantial quantum tunneling through the insulating barrier.	Low values are desirable for IR rectennas and can be achieved using ultrathin, low-bandgap barriers with optimized barrier heights.

**Table 2:** Summary of recent achievements in the field of MIM diodes [3].

Material	Cut-off Frequency	Thickness	J_ON_	*f*_ASYM_	*f*_NL_	*f*_RES_ (V^−1^)	Zero Bias *f*_RES_ (V^−1^)
Cu (100 nm)-CuO-Au (100 nm) (0.0045 μm^2^) [9]	28.3 THz	CuO (0.7 nm) Au/Cu (100 nm)	-	-	-	6	4
Ti-TiO_2_-Al (21,287 µm^2^) [10]	Up to 150 THz	TiO_2_ (9 nm)	10^−1^ A/cm^2^	-	6.5	18	-
Ti-TiO_2_-Pt (21,287 µm^2^) [10]	Up to 150 THz	TiO_2_ (9 nm)	10^−0^ A/cm^2^	-	15	15	-
Nb/Nb_2_O_5_/Pt [11]	Up to 150 THz	Nb_2_O_5_ (15 nm)	-	1500	4	20	-
Nb/Nb_2_O_5_/Cu [11]	Up to 150 THz	Nb_2_O_5_ (15 nm)	-	1500	8	20	-
Nb/Nb_2_O_5_/Ag [11]	Up to 150 THz	Nb_2_O_5_ (15 nm)	-	1500	8	20	-
Nb/Nb_2_O_5_/Au [11]	Up to 150 THz	Nb_2_O_5_ (15 nm)	-	1500	8	20	-
Au/Al_2_O_3_/Pt [12]	Up to 28.3 THz	Al_2_O_3_ (1.4 nm) Au/Pt (100 nm)	-	-	6	-	10
Ni-NiO-Ag (3.1 × 10^−4^ µm^2^) [13]	Up to 343 THz	NiO (6 nm)	-	5	3	8.5	5.8
Pt-SiCl_3_-(CH_2_)_17_-CH_3_ -Ti (100 μm^2^) [14]	Up to 150 THz	SiCl_3_-(CH_2_)_17_-CH_3_ (2.23 nm)	-	117.8	6.8	20.8	8.0
Nb/TiO_2_/Pt [15]	Up to 30 THz	TiO_2_ (13 nm)	-	80	3.5	-	-
Nb/Nb_2_O_5_/Ni [15]	Up to 150 THz	Nb_2_O_5_ (15 nm) Nb/Ni (90–100 nm)	1 × 10^−10^ A/cm^2^	396.5	7.1	8.5	-
Nb/Nb_2_O_5_ (15 nm)/Au [15]	Up to 150 THz	Nb_2_O_5_ (15 nm) Nb/Au (90–100 nm)	-	1430.8	8.0	7.0	-
SrTiO_3_ (STO)/Al_2_O_3/_SrTiO_3_ (STO) [16]	Up to RF	-	5 × 10^−9^ A/cm^2^	-	-	-	-
Cu-CuO-Cu (2 × 2 μm^2^) [17]	Up to 150 THz	CuO (2 nm) Cu (100 nm)	-	-	-	4.497	-
Pt/Al_2_O_3_/Al [18]	Up to 150 THz	Al_2_O_3_ (6 nm) Pt/Al (100 nm)	-	110 for AP-CVD 30 for PEALD	6 for AP-CVD 30 for PEALD	9 for AP-CVD 22 for PEALD	-
Al-Al_2_O_3_-Au [19]	Up to 60 THz	Al/Au (65 nm)	4.0 μA/cm^2^	-	-	14.46	-
Al-Al_2_O_3_-Cr [20,21]	Up to 28.3 THz	Al_2_O_3_ (3 nm) Al/Cr (100 nm)	2 × 10^−4^ A/cm^2^	-	3.1	-	-
This work NbS_2_-Sc_2_O_3_-Mo_2_C	Up to 28.3 THz	Sc_2_O_3_ (2 nm)-NbS_2_/Mo_2_C (100 nm)	1.05 × 10^2^ A/cm^2^	2.1 × 10^4^	1	7 at 0.1 V	10
This work VSe_2_/LaOF/VN	Up to 28.3 THz	LaOF (2 nm)-VSe_2_/VN (100 nm)	1.60 × 10^2^ A/cm^2^	1.4 × 10^4^	1	7 at 0.1 V	10
This work TaS_2_/LaOBr/V	Up to 28.3 THz	LaOBr (2 nm)-TaS_2_/V	2.2 × 10^2^ A/cm^2^	2.5 × 10^5^	1	7 at 0.1 V	10

**Table 3:** Design parameters of the NbS_2_–Sc_2_O_3_–Mo_2_C MIM diode.

Parameter	Value
Work function of NbS_2_ $\left(\Psi_{1}\right)$ [27]	4.70 eV
Work function of Mo_2_C $\left(\Psi_{2}\right)$ [28]	3.4 eV
Electron affinity of Sc_2_O_3_ $\left(\chi \right)$ [29]	3.0 eV
Bandgap of Sc_2_O_3_ [29]	6 eV
Left barrier height $\left(\Phi_{L}=\Psi_{1}-\chi \right)$	1.70 eV
Right barrier height $\left(\Phi_{R}=\Psi_{2}-\chi \right)$	0.4 eV
Work function difference $\left(\Delta \Psi \right)$	1.30 eV
Breakdown voltage V_BD_$\;\approx 2\left(\Psi_{1}-\Psi_{2}\right)$	2.60 V
Insulating layer thickness d	2 nm

**Table 4:** Design parameters of the VSe_2_-LaOF-VN MIM diode.

Parameter	Value
Work function of VSe_2_ $\left(\Psi_{1}\right)$ [30]	5.60 eV
Work function of VN $\left(\Psi_{2}\right)$ [31]	4.35 eV
Electron affinity of LaOF $\left(\chi \right)$ [32]	4.0 eV
Bandgap of LaOF [32]	6 eV
Left barrier height $\left(\Phi_{L}=\Psi_{1}-\chi \right)$	1.60 eV
Right barrier height $\left(\Phi_{R}=\Psi_{2}-\chi \right)$	0.35 eV
Work function difference $\left(\Delta \Psi \right)$	1.25 eV
Breakdown voltage V_BD_$\;\approx 2\left(\Psi_{1}-\Psi_{2}\right)$	2.50 V
Insulating layer thickness d	2 nm

**Table 5:** Design parameters of the TaS_2_/LaOBr/V MIM diode.

Parameter	Value
Work function of TaSe_2_ $\left(\Psi_{1}\right)$ [33]	5.90 eV
Work function of V $\left(\Psi_{2}\right)$ [34]	4.3 eV
Electron affinity of LaOBr $\left(\chi \right)$ [35]	4.0 eV
Bandgap of LaOBr [35]	5.3 eV
Left barrier height $\left(\Phi_{L}=\Psi_{1}-\chi \right)$	1.90 eV
Right barrier height $\left(\Phi_{R}=\Psi_{2}-\chi \right)$	0.30 eV
Work function difference $\left(\Delta \Psi \right)$	1.6 eV
Breakdown voltage V_BD_$\;\approx 2\left(\Psi_{1}-\Psi_{2}\right)$	3.20 V
Insulating layer thickness d	2 nm

**Table 6:** Summary of key performance parameters for the three MIM diode configurations.

Parameter	NbS_2_-Sc_2_O_3_-Mo_2_C	VSe_2_/LaOF/VN	TaS_2_/LaOBr/V
Current Density (J-V) at zero bias	1.05 × 10^2^ A/cm^2^	1.60 × 10^2^ A/cm^2^	2.2 × 10^2^ A/cm^2^
Current I(V) at zero bias	9.5 × 10^−10^ A	1.44 × 10^−9^ A	2 × 10^−9^ A
Forward Current (IF)	3.0 × 10^−6^ A	5.02 × 10^−6^ A	7 × 10^−6^ A
Reverse Current (IR)	1.47 × 10^−10^ A	3.60 × 10^−10^ A	3.5 × 10^−11^ A
I_ON_/I_OFF_	20,408	13,944	200,000
Asymmetry	2.1 × 10^4^	1.4 × 10^4^	2 × 10^5^
Asymmetry at ±0.3 V	2680	2580	3300
Nonlinearity at zero bias	1	1	1
Responsivity at zero bias	10 V^−1^	10 V^−1^	10 V^−1^
Responsivity at 0.1 V	7 V^−1^	7 V^−1^	7 V^−1^
Zero-bias resistance (R_0_)	5.3 × 10^5^ Ω	3.50 × 10^5^ Ω	2.5 × 10^5^ Ω

## Data Availability

The original contributions presented in this study are included in the article. Further inquiries can be directed to the corresponding author.

## Abbreviations

The following abbreviations are used in this manuscript:

LWIR	Long-Wave Infrared
MIM	Metal–Insulator–Metal
IRAs	Infrared Rectifying Antennas
I–V	Current–Voltage
J-V	Current Density–Voltage
FOMs	Figures of Merit
TOV	Turn-On Voltage
THz	Terahertz (10^12 Hz)
RF	Radio Frequency
εr	Relative Permittivity (Dielectric constant)
Φ	Barrier height
χ	Electron affinity of the insulator material
d	Tunnel Barrier Thickness
V	Bias Voltage
R_0_	Zero-Bias Resistance
f_ASYM_	Asymmetry Ratio (or asymmetry factor)
f_NL_	Nonlinearity Factor
f_RES_	Responsivity
R_D_	Differential Resistance
ΔΨ	Work Function Difference between electrodes
V_BD_	Breakdown Voltage
A	Effective Junction Area
